# Supplementary material for: Zambian Peer Educators for HIV Self-Testing (ZEST) study: rationale and design of a cluster randomised trial of HIV self-testing among female sex workers in Zambia
Source: BMJ Open. 2017 Apr 20;7(4):e014780. doi: 10.1136/bmjopen-2016-014780 (PMC5775452; doi:10.1136/bmjopen-2016-014780)
Supplement: Author's manuscript [file bmjopen-2016-014780.draft_revisions.pdf]

BMJ Open

The Zambian Peer Educators for HIV Self-Testing (ZEST) Study: Rationale and design of a cluster randomized trial of HIV self-testing among female sex workers in Zambia

Journal:	*BMJ Open*
Manuscript ID	bmjopen-2016-014780
Article Type:	Protocol
Date Submitted by the Author:	18-Oct-2016
Complete List of Authors:	Oldenburg, Catherine; University of California San Francisco, Francis I Proctor Foundation<br>Ortblad, Katrina; Harvard T.H. Chan School of Public Health, Global Health and Population<br>Chanda, Michael; John Snow, Inc<br>Mwanda, Kalasa; John Snow, Inc<br>Nicodemus, Wendy; John Snow, Inc<br>Sikaundi, Rebecca; John Snow, Inc<br>Fullem, Andrew; John Snow, Inc<br>Barresi, Leah; Harvard T.H. Chan School of Public Health, Epidemiology<br>Harling, Guy; Harvard School of Public Health, Department of Global Health and Population<br>Bärnighausen, Till; University of Heidelberg
**Primary Subject Heading**:	HIV/AIDS
Secondary Subject Heading:	Global health, Health services research
Keywords:	female sex workers, HIV testing, HIV self-testing, Sub-Saharan Africa, HIV & AIDS < INFECTIOUS DISEASES

SCHOLARONE™
Manuscripts

The Zambian Peer Educators for HIV Self-Testing (ZEST) Study: Rationale and design of a cluster randomized trial of HIV self-testing among female sex workers in Zambia

Catherine E. Oldenburg[1,2], Katrina F. Ortblad[3], Michael M. Chanda[4], Kalasa Mwanda[4], Wendy Nicodemus[4], Rebecca Sikaundi[4], Andrew Fullem[5], Leah G. Barresi[1], Guy Harling[3], Till Bärnighausen[3,6]

[1]Department of Epidemiology, Harvard T.H. Chan School of Public Health, Boston, MA, USA
[2]Francis I. Proctor Foundation, University of California, San Francisco, USA
[3]Department of Global Health and Population, Harvard T.H. Chan School of Public Health, Boston, MA, USA
[4]John Snow, Inc, Lusaka, Zambia
[5]John Snow, Inc, Boston, MA, USA
[6]Africa Center for Population Health, Mtubatuba, South Africa

Corresponding Author
Catherine E. Oldenburg, ScD, MPH
513 Parnassus Ave, Room S334
San Francisco, CA 94143-0412
catherine.oldenburg@ucsf.edu

Word Count: 4,183
Abstract Word Count: 328
Tables: 1
Figures: 3
References: 37

For peer review only

For peer review only - http://bmjopen.bmj.com/site/about/guidelines.xhtml

ABSTRACT

Background. HIV self-testing (HIVST) may be an alternative to existing available testing services for female sex workers (FSW), but to date little evidence exists for its implementation. Here, we describe the rationale and design of a cluster randomized trial designed to inform implementation and scale-up of HIVST programs for FSW in Zambia.

Methods. The Zambian Peer Educators for HIV Self-Testing (ZEST) study is a three-arm cluster randomized trial taking place in three towns in Zambia. Participants (N=900) are eligible if they are women who have exchanged sex for money or goods in the previous 3 months, are HIV negative or status unknown, have not tested for HIV in the previous 3 months, and are at least 18 years old. Participants are recruited by peer educators working in their communities. Participants are randomized to one of three arms: 1) direct distribution, in which they receive an HIVST from the peer educator directly; 2) fixed distribution, in which they receive a coupon with which to collect the HIVST from a distribution point (drug store or health post); or 3) referral to existing HIV testing services only. Participants are followed at one and four months following distribution of the first HIVST, and individuals in the HIVST arms are offered a second HIVST at approximately 3 months. The primary endpoint is HIV testing in the past month measured at the one- and four-month visits. Secondary endpoints include awareness of HIV status measured at four months, use of the HIVST for those in HIVST arms, and linkage to care.

For peer review only

Discussion. This trial will provide robust evidence of the efficacy of HIVST for improving HIV testing coverage among FSW, and will allow for assessment of both whether they are acceptable to FSW and if FSW can access them at common distribution points within the health system.

ClinicalTrials.gov: NCT# NCT02827240

Keywords: HIV testing; HIV self-testing; female sex workers; cluster randomized trial

Strengths and limitations of this study

- This study will be one of the first studies reporting an HIV self-testing intervention among female sex workers (FSW) and will enroll a large sample size from three towns in Zambia
- This study will allow for assessment of both whether HIV self-test kits are used by participants as well as if they are accessible via distribution points within the existing health system
- We utilize several innovative measurement schemes that may reduce social desirability bias in the reporting of outcomes
- This study does not rely on clinic or other venue-based sampling, and thus may access populations of FSW who have had reduced access to HIV testing
- Limitations of this study include the self-reported nature of many of the outcomes, which may lead to social desirability bias

For peer review only

INTRODUCTION

Female sex workers (FSW), defined as women who exchange sex for money or goods, bear a disproportionate burden of the HIV epidemic globally.[1,2] Global HIV prevalence among FSW has been estimated at nearly 12%, with HIV prevalence rising to more than 30% for FSW living in countries with medium and high background HIV epidemics.[1] In Sub-Saharan Africa in 2011, an estimated 17.8% of all infections in women were attributed to engagement in sex work.[3] Vulnerability to HIV among FSW occurs at multiple levels[4], which potentiates the HIV epidemic in this population.[2,5] Determinants of HIV among FSW occur at the individual level (behavioral and biological factors, such as sexually transmitted infections), dyad and partner level (such as power dynamics within relationships), work environment level (organizational structures where individuals work, including venue policies and availability of condoms in venues)[6], and community and macrostructural levels (including community structure and organization and legal and policy environments).[2] Furthermore, across all levels, the influence of stigma and discrimination can affect access to HIV prevention and other healthcare services.[7-10]

In 2014, the Joint United Nations Programme on HIV and AIDS (UNAIDS) put forth a new global target for controlling and ultimately ending the HIV epidemic.[11] The 90-90-90 target is a three-part treatment target that aims to have 90% of people living with HIV aware of their status, 90% on treatment, and 90% virally suppressed by the year 2020, ultimately resulting in viral suppression for 73% of HIV-infected individuals worldwide. Such a strategy requires regular HIV testing for individuals who are at ongoing risk of HIV, including FSW. However, significant

For peer review only - http://bmjopen.bmj.com/site/about/guidelines.xhtml

gaps remain in HIV testing coverage in Sub-Saharan Africa.[12] Given the large burden of HIV among FSW and the unique barriers to HIV prevention faced by this population, evidence of strategies that are specifically designed for this population are urgently needed.

Oral HIV self-testing (henceforth, HIVST) is an alternative to clinic or other healthcare provider testing (e.g., home-based HIV testing and counseling) that may overcome some barriers to traditional HIV testing strategies for some populations.[13-16] HIVST has been shown to be acceptable in a variety of populations globally[13], but very little data exist on the acceptability and feasibility of HIV self-testing among FSW.[14] For FSW specifically, HIVST may offer several advantages over traditional HIV testing strategies that may improve status knowledge, and, ultimately progress towards the 90-90-90 target. HIVST allows for an individual to test in privacy and on her own time. Two major barriers to testing for FSW in regular clinics include stigma and timing. Healthcare provider stigma is a major deterrent to engagement in care for stigmatized populations.[17] It can be difficult for FSW to test for HIV or access treatment because healthcare workers might refuse to serve them or require them to identify sexual partners. Furthermore, even in the absence of stigma, existing clinics may not offer HIV testing services during hours which are feasible for FSW, who often sleep during the day and work during the night. HIVST can overcome each of these limitations by allowing a woman to test at a time and a place that is convenient and acceptable to her.

For peer review only - http://bmjopen.bmj.com/site/about/guidelines.xhtml

For peer review only

A rapid oral HIVST has been approved in the United States, which uses fluid from the oral mucosa and is read in 20 minutes.[18] The test, OraQuick (OraSure Technologies Inc, Bethlehem, PA, USA), has a sensitivity of 91.7% and a specificity of 99.9% among untrained users.[19] All individuals who test positive with this test are encouraged to get a blood-based confirmatory test. Among men who have sex with men (MSM) in Seattle, a randomized controlled trial demonstrated a significant increase in HIV testing coverage among individuals receiving HIVST.[20] Although HIVST may be an acceptable alternative to regular HIV testing for FSW, to date minimal evidence of the use of HIV self-testing among FSW exists.[21] The acceptability, accuracy, uptake, and efficacy of HIVST among FSW remains largely unknown.

Here, we describe a cluster randomized controlled trial that addresses this critical gap in the HIVST evidence for FSW. In the trial, we test two health service delivery mechanisms, a direct distribution system where a FSW participant is directly given an HIVST by a peer, and a fixed distribution system where the FSW participant collects the HIVST from a fixed point such as a pharmacy or health post. The trial seeks to determine the efficacy of each HIVST delivery mechanism, compared to standard of care, for improving HIV testing frequency and knowledge of HIV status.

METHODS/DESIGN

Theory of Change

For peer review only - http://bmjopen.bmj.com/site/about/guidelines.xhtml

The intervention tested in the ZEST study, and data collected throughout the course of the study, was guided *a priori* by a theory of change developed through mental models ad deductive development.[22] Briefly, we theorized that the distribution of HIV self-test kits via peer educators would lead to improved status knowledge by reducing barriers to HIV testing such as stigma or logistical barriers such as hours of operation. This would lead to improved uptake of HIV testing, which would lead to improved knowledge of status and ultimately reduce time to linkage to care.

Study Design

This is a non-blinded cluster randomized trial. Individuals are enrolled as members of a peer educator group, with a target enrollment of five FSW per peer educator. The peer educator-FSW group is randomized in a 1:1:1 fashion to one of the three study arms: 1) peer educator distribution of HIVST directly to participants (*direct distribution*), 2) peer educator distribution of a coupon valid for an HIVST at a fixed distribution point (*fixed distribution*), or 3) standard of care. Participants will be followed for four months from enrollment, with follow-up assessments at Months 1 and 4 (Figure 1).

Specific Aims

The specific aims of this trial are (1) establish the impact of two different distribution mechanisms (direct and fixed distribution) on HIV testing coverage; (2) establish the effect of the two distribution mechanisms on HIV status knowledge;

For peer review only - http://bmjopen.bmj.com/site/about/guidelines.xhtml

(3) measure HIV self-testing uptake by distribution mechanism; and (4) measuring linkage to care by the different mechanisms. The trial design will allow for comparison of the two distribution mechanisms to each other as well as to standard HIV testing.

Study Oversight

An independent Scientific Oversight Committee (SOC) oversees the data collected as part of this study. The SOC contains members with expertise in HIV epidemiology, statistics, ethics, and female sex work. The SOC reviews any reports of serious adverse events during the course of the trial, and will convene a meeting after approximately half of the subjects have reached their one-month visit. Due to the short duration of the study, there are no formal statistical stopping rules.

Setting

This study is taking place in three border and transit towns in Zambia: Chirundu, Livingstone, and Kapiri (Figure 2). Chirundu and Livingstone are located on the Zambia-Zimbabwe border, and are a major transportation points for people and goods. Kapiri is north of the capital, Lusaka, and is a transit hub, with a large weigh station where many truckers stop for the night or longer. Study headquarters and coordination for the three sites is located in the capital, Lusaka.

Recruitment

For peer review only - http://bmjopen.bmj.com/site/about/guidelines.xhtml

Participants are recruited by peer educators. The peer educators are FSW themselves who were recruited by study staff and who have undergone a two-day training in study procedures and HIV self-testing. Interested potential participants are referred to study staff after the peer educator describes the study to them. A research assistant then conducts a phone screening to determine whether or not the participant is eligible. A formal eligibility assessment is then done in person with the research assistant and eligible individuals complete an informed consent procedure. Written informed consent is obtained from each participant.

Inclusion and Exclusion Criteria

Complete inclusion and exclusion criteria are summarized in Table 1. Participants must be women 18 years of age or older who self-report exchanging sex (including vaginal, anal, and/or oral) for money or goods in the last month.

Individuals who are currently residing in the PopART study catchment area[23] in Livingstone will be excluded from this study, in addition to any individual who reports concomitant enrollment in another HIV prevention study. The PopART study includes a household HIV testing program, which would bias outcomes in this study if individuals were being regularly tested as part of another HIV prevention study.

Randomization

Peer educator groups (the women recruited by a particular peer educator) are randomized as a unit. Group randomization occurs after all members of the

For peer review only

group have been enrolled in the study and completed their baseline questionnaire. Groups are randomized to one of the three study arms using a computer generated randomization list in random blocks of 3, 6, and 9 and stratified by study site (Livingstone, Chirundu, and Kapiri). Randomized study arm assignments are placed in an opaque envelope, and once all participants in a peer educator group have been enrolled, the research assistant obtains the envelope from the research coordinator or the site coordinator and opens the envelope with the peer educator.

Study Procedures

An overview of the study procedures and activities is summarized in Table 1 and Figure 3.

<u>Peer Educator Visits</u>

In all study arms, peer educators will meet with participants at least four times (approximately monthly) over the course of the study. The first peer educator intervention visit will be a group-based visit, and includes simple HIV prevention information, information on where to go for HIV testing, and condom provision. In the HIVST arms, peer educators will provide a brief overview of how to use the self-test kits, who to contact if participants have problems or concerns, and the importance of as well as how to obtain additional care, including confirmatory testing and linkage to care. Follow-up visits with peer educators are comprised of brief check-in visits, during which peer educators will distribute condoms, discuss any issues participants have with the HIVST kits or other elements of the study, and

For peer review only - http://bmjopen.bmj.com/site/about/guidelines.xhtml

screen for adverse events including intimate partner violence. Between visits, peer educators will be available should participants need help with any issue relating to safe sex, HIV, violence, or any other elements of the study.

Direct Distribution Arm

In the direct distribution arm, peer educators will directly distribute HIVST kits to participants during the first intervention visit. The HIVST kit consists of a single OraQuick in-home HIV self-test and a set of instructions translated into English, Nyanja, and Bemba. The instructions are a step-by-step pictorial and written guide for using the test and interpreting the results.

Peer educators offer a second HIVST kit to participants in the direct distribution arm at the fourth intervention visit. There is no HIV status requirement for distribution of a second test. The second test is distributed at the last peer educator visit to minimize contamination of study arms.

Fixed Distribution Arm

In the fixed distribution arm, peer educators give participants a coupon which they can exchange for an HIVST kit at a participating fixed distribution facility. These facilities include drug stores and health posts that have agreed to participate in the study and have received basic training on HIVST. Staff at the fixed distribution points collect the coupons when they are exchanged for an HIVST kit and give them to research staff on a weekly basis. These coupons include a bar code that is scanned by study staff to record which participants collected the HIVST kit.

For peer review only - http://bmjopen.bmj.com/site/about/guidelines.xhtml
For peer review only

Peer educators offer a second coupon to participants at the fourth intervention visit to individuals, similar to the direct distribution arm. As with the first HIVST kit, participants will use a coupon to obtain the second HIVST kit at a participating fixed distribution point.

Standard of Care Arm

In the standard of care arm, peer educators give participants information about existing HIV testing services where participants can go for testing. In Livingstone, Chirundu, and Kapiri, available options generally include government facilities, non-profit faith-based facilities, and private facilities. While clinics designed specifically for female sex workers were previously in operation in these cities, funding constraints forced these facilities to close in 2015. The information that participants receive about HIV testing facilities is customized to the city of their recruitment, and identical information is given to participants in each of the three arms.

In all study arms, participants will receive a linkage to care card from their peer educator that they will be requested to bring if they seek confirmatory testing and HIV care.

Data Collection

Baseline Survey

At baseline, all participants complete a quantitative questionnaire administered by a research assistant. Data are collected directly on a tablet in the

For peer review only - http://bmjopen.bmj.com/site/about/guidelines.xhtml

field. All assessments are conducted in a private room at a time and location that is convenient for the participant. The baseline survey covers broad topics including demographics and household composition, sex work and professional history, reproductive health and contraception, sexual behaviors, HIV testing history, attitudes towards HIV self-testing, access to healthcare, and measures of substance use[24], depression[25], social support, self-efficacy[26], and empowerment.[27]

Follow-up Surveys

Follow-up surveys will be conducted at one month and four months from the baseline visit (defined as the first intervention visit with the peer educator). Participants in all arms of the study will be asked about their recent HIV testing history, sexual behaviors, access to healthcare, and linkage to care. In the HIVST arms of the study, participants will additionally be asked about their experiences with the HIVST kit, including if they used it and details about their experience using the test, as well as their experience with the peer educator. Participants in all arms are asked again at one and four months about HIVST acceptability and perceived likelihood of using such tests in the future.

Qualitative Interviews

Approximately 5% of the sample (15 participants per arm, 45 in total) are randomly selected at baseline to participate in individual qualitative in-depth interviews. During baseline screening, a computer generated program randomly prompts the research assistant to ask the participant if they would be interested in

For peer review only

participating in the qualitative phase of the study. Individual in-depth interviews are conducted by trained research assistants at baseline and at the four-month follow-up visit. Baseline interviews explore community norms related to HIV testing, multilevel (including individual, structural, and interpersonal) barriers to accessing HIV testing and other healthcare, experiences in sex work, and baseline perceptions of HIV self-testing. At follow-up, qualitative interviews focus on experiences using the HIV self-test for individuals who are in one of the two intervention arms, and individuals in all arms will be asked about their perceptions of the HIV self-test and how HIVST might be implemented at scale. In all arms at follow-up, the interviews will explore empowerment and self-efficacy related to HIV testing and HIV status disclosure.

Endpoint Measurement

Study endpoints are summarized in Table 3. The primary endpoint for the study is the proportion of participants who tested in the previous month as assessed at the one-month follow-up visit. Secondary endpoints include testing at four months, awareness of HIV status, HIVST kit use (restricted to the HIVST arms), linkage to care, and adverse events.

Recent HIV Testing

Recent HIV testing is assessed by self-report at the one-month and four-month visits. Participants are asked when their last HIV test was, and then asked where their test was to determine if their last test was an HIVST or other.

For peer review only - http://bmjopen.bmj.com/site/about/guidelines.xhtml

HIV Status Knowledge

Correct reporting of HIV status via self-report is subject to social desirability bias. To accurately measure whether an individual knows their HIV status, they must both correctly report knowledge of their HIV status and be willing to take an HIV test. To overcome some of the bias in ascertaining self-reported HIV status, we use a three-step approach. First, participants are asked if they know their current HIV status. They are then asked to tell the research assistant their current HIV status, and are told to take their best guess if they do not know. Participants are then asked to take a rapid HIV test, and are counseled that this test is entirely voluntary. To reduce bias in self-reporting HIV status, participants are told that they will receive a small gift (worth approximately USD$1) if they correctly report their status. However, after the assessment, all participants will receive the gift regardless of their reported status, thus the cash transfer is perceived as conditional but is actually unconditional for ethical reasons. Pre- and post-test counseling will be available to all individuals who participate in the HIV status assessment. This assessment is performed only at four months.

For peer review only

HIVST Kit Use

Actual use of the HIVST kit may also be subject to social desirability bias. Participants may want to report that they used the HIVST kit when they did not for many reasons, including desire to please the researchers, a sense of obligation, or misperception about what it means to use the test.[28] As an attempt to reduce bias in

For peer review only - http://bmjopen.bmj.com/site/about/guidelines.xhtml

ascertainment of actual use of the HIVST kits, research assistants will ask participants to buy back any unused HIVST kits at the end of the study with the justification that the study has now reached completion. Participants will be offered approximately USD$1 in exchange for any unused test kits in their original packaging. Prior to this offer, participants are not told that researchers will buy back tests at the end of the study (this information is also not included in the informed consent). To avoid gossip among study participants, the first buy-back will not happen until at least one month after the last test kit has been distributed (with the assumption that all participants would have used the test kit at that point if there were planning to).

Adverse Events

Adverse events are monitored and documented throughout the study. The primary adverse events of concern are psychological harm as a result of use of the HIV self-test (for example, if participants test positive for HIV and are not prepared for the results). Although UNAIDS has recently indicated no evidence of serious adverse events related to HIV self-testing (i.e., self-harm, violence, or human rights violations)[29], occurrence of any such events are reported all principal investigators and the Scientific Oversight Committee (SOC) within 24 hours of study staff becoming aware of them. Other adverse events include coercive testing or unintentional or unauthorized disclosure of HIV status, which are similarly reported within 24 hours.

For peer review only - http://bmjopen.bmj.com/site/about/guidelines.xhtml

Statistical Analysis

The primary analysis will be intention-to-treat (ITT). The primary analysis for the quantitative data will involve a mixed-effects multilevel logistic regression model to account for clustering by peer educator group and study site (participants nested in peer educators nested in study sites). These models will include an indicator term for study arm (fixed distribution, direct distribution, or control). Outcomes, including past-month HIV testing and correct knowledge of status at four months, will be modeled as dichotomous variables.

Sample Size Considerations

The sample size calculation was based on the primary endpoint, testing for HIV in the past month assessed at the one-month visit, and the secondary endpoint, proportion of participants who correctly know their status at the four month visit. Power calculations were performed using methods for cluster randomized trials, with clustering by peer educator identity. We assume that 50% of participants will have tested in the past month in the control arm based on previous estimates of HIV testing behavior among FSW in Livingstone and Chirundu.[30,31] We estimate that in the control group, approximately 70% of participants will correctly identify their HIV status, assuming that the majority of those whose status is unknown will report that they are negative, but that approximately half of these individuals will actually be positive. Assuming 20% loss to follow-up and with 50 peer educators (clusters) per arm, a sample size of 300 participants per arm (900 total) will yield 89% power to detect a risk ratio of 1.30 for recent testing and 94% power to detect a risk ratio

For peer review only - http://bmjopen.bmj.com/site/about/guidelines.xhtml

of 1.20 for correct status knowledge, assuming a type I error probability of 0.05 and an intracluster correlation (ICC) of 0.03.

DISCUSSION

Despite disproportionate burden of HIV and complex, multilevel barriers that can restrict access to HIV prevention, there remain relatively few HIV prevention interventions developed specifically for FSW in Sub-Saharan Africa. The overarching goal of the ZEST trial is to provide robust evidence for the development of policy for implementation and scale-up of HIVST in Zambia with a specific focus on ensuring this technology is accessible to FSW. Working in concert with the Ministry of Health in Zambia and the World Health Organization, this trial was specifically designed to fill a crucial gap in the evidence by answering two questions: 1) is HIVST an acceptable alternative to standard HIV testing for FSW, and 2) will FSW access HIVST.

Question One is addressed through the direct distribution arm. In this arm, we will be able to assess whether individuals directly given a test actually use the test. By directly giving individuals the test, we are assessing best-case scenario uptake, where there as few barriers as possible for individuals to access the test kit. The uptake results from this arm of the study will give robust evidence on whether HIVST is an acceptable alternative to standard and currently-available HIV testing services. Coupled with qualitative data, we will be able to measure both the uptake of HIVST, but also participant attitudes towards HIVST. This will lead to an in-depth

For peer review only - http://bmjopen.bmj.com/site/about/guidelines.xhtml

understanding of why participants did or did not use the test, and how participants view the role of HIVST for HIV testing in the future.

Although uptake and acceptability of HIVST is an important piece of evidence for HIVST policy, the design of this study allows for measurement of a more realistic scenario, in which participants must go to a physical distribution point to collect their kit. Question Two is thus addressed through the fixed distribution arm. This arm will measure the degree to which barriers to acquiring the HIVST kit in the store affect acquisition and use of the test. This will be tested by implementing the project within existing drug stores and health posts, without changing the hours or geographical locations of existing services. The direct and fixed distribution arms will therefore give two complementary pieces of information – first, are participants using the test kits, and second, are they able to access them independently.

A major strength of this study is that it does not rely on clinic-based sampling or recruitment. A potential benefit of HIVST over existing HIV testing services is that it may allow individuals who are not currently engaged in care or who have never sought HIV testing because of barriers to accessing care to test for HIV. HIVST may therefore be a gateway to care for individuals who have previously been difficult to engage in care. By working through peer educators, rather than in clinics, we will access a different subset of the population. Peer educator programs have been used in a variety of settings with different sex worker populations to increase HIV prevention knowledge.[32-37] In many cases, peer educators have unique access to and rapport with FSW. This study therefore is expected to enroll a diverse population of FSW that is likely more generalizable than a clinic-based sample,

For peer review only - http://bmjopen.bmj.com/site/about/guidelines.xhtml

while simultaneously allowing access to populations that may not currently access care.

A major concern with HIVST is the potential for intimate partner violence, for example if sexual partners find out about HIV status through the test. All participants will be screened for intimate partner violence at baseline, one, and four months. Furthermore, during follow-up peer education visits, peer educators will screen for intimate partner violence, and they will also be available between visits should any issues come up. Finally, a 24-hour telephone line will be available to all participants for any issues, including intimate partner violence. All reports of intimate partner violence will be reviewed to determine if they are related to HIVST or trial participation. In this study, we do not offer HIVST for FSW to test with their clients. While it is possible that this could be a benefit for some participants, it could also put some at increased risk of violence. We will collect detailed quantitative and qualitative data about with whom participants used the test, if anyone. The results of this study are expected to yield evidence of the effect of HIVST on intimate partner violence experiences, however we do not expect this study to provide evidence on intimate partner violence in the context of partner testing.

Another concern with HIVST is the potential for depression, anxiety, or suicidality as a result of learning one's HIV status. Structured interviews will contain screening questions for depression, and peer educators will perform a brief screening on depression, anxiety, and self-harm at each visit. Finally, participants will be instructed to call the hotline should they need to discuss their HIV result with anyone or receive counseling.

For peer review only - http://bmjopen.bmj.com/site/about/guidelines.xhtml

In conclusion, the results of this study are expected to inform policy aimed at implementation and scale-up of HIVST for FSW in Zambia. This study will begin to address critical gaps in the literature describing evidence of HIV testing interventions for FSW in Southern Africa. We expect the results of this trial will have a direct and measureable impact on the availability of HIVST in Zambia.

For peer review only

For peer review only - http://bmjopen.bmj.com/site/about/guidelines.xhtml

DECLARATIONS

Ethical Approval

Ethical approval was obtained for the Zambian Peer Educators for HIV Self-Testing Study from the Harvard T.H. Chan School of Public Health Institutional Review Board in Boston, Massachusetts and the ERES Converge Institutional Review Board in Lusaka, Zambia. Written informed consent is obtained from all participants.

Availability of Data and Material

Upon completion of the trial, de-identified data will be available through the International Initiative for Impact Evaluation (3ie)'s public data access repository on Dataverse.

Competing Interests

None to report.

Funding

The Zambian Peer Educators for HIV Self-Testing Study is funded by the International Initiative for Impact Evaluation (3ie). CEO was supported in part by the National Institute on Drug Abuse T32-DA013911 and the National Institute of Mental Health R25-MH083620. KFO was supported in part by the National Institute of Allergy and Infectious Disease T32-AI007535. TB was supported in part by the National Institute of Child Health and Human Development R01-HD084233 and the National Institute of Allergy and Infectious Diseases R01-AI124389 and R01-

For peer review only - http://bmjopen.bmj.com/site/about/guidelines.xhtml

AI112339. The study sponsor is not involved in study design, collection, management, analysis or interpretation of the data, of writing reports or in the decision to submit the report for publication.

Authors' Contributions

Conceived and designed study: CEO, KFO, MMC, KM, WN, RS, AF, LGB, GH, TB. Created tables/figures: CEO, KFO, LGB. Wrote first draft of manuscript: CEO, KFO, MMC. Read and critically revised manuscript: CEO, KFO, MMC, KM, WN, RS, AF, LGB, GH, TB.

Acknowledgements

We gratefully acknowledge feedback on and conversations related to our study design from Anna Heard, Annette Brown, and Nancy Diaz.

For peer review only

For peer review only - http://bmjopen.bmj.com/site/about/guidelines.xhtml

Table 1. Study inclusion/exclusion criteria

Inclusion criteria	***Exclusion criteria***
• 18 years or older at enrollment	• Less than 18 years at enrollment
• Reports exchanging sex (vaginal, oral, and/or anal) for money or goods at least once in the past month	• Has not exchanged any form of sex in the past month
• Self-reported HIV negative and no recent HIV test (<3 months) OR HIV status unknown	• Self-reported to be living with HIV<br>• Self-reported HIV negative but tested in the within the last 3 months
• Permeant residence in the study town of enrollment (Livingstone, Chirundu, Kapiri)	• Planning to move out of the geographical area within 4 months<br>• Living in the PopART catchment area (Livingstone only)
• Willing to participate in peer education sessions and study assessments over 4-month study period	• Meets inclusion criteria but does not wish to participate
	• Concurrently participating in another HIV prevention study

For peer review only - http://bmjopen.bmj.com/site/about/guidelines.xhtml

Table 2. Outline of study visits

Study Time Point	*Visit By*	*Activities*
**Recruitment**	Peer Educator	• Peer educator discusses study with potentially eligible participant and refers them to study staff
**Enrollment**	Research Assistant	• Assessment of eligibility<br>• Informed consent (if eligible)<br>• Baseline quantitative survey<br>• Baseline qualitative survey (~5%)
**Randomization**	Research Assistant	• Randomization of peer educator and their cohort of participants once all group members are enrolled
**Intervention Visit 1 (Week 0)**	Peer Educator	• Group-based intervention<br>• HIV prevention counseling and distribution of condoms<br>• Training on HIV self-test use (in HIVST arms)<br>• Distribution of HIV self-tests or coupons (in HIVST arms)
**Intervention Visit 2 (Week 2-3)**	Peer Educator	• Screening for adverse events<br>• Discussion of any difficulty with HIV self test use (in HIVST arms)<br>• Referral to care and standard HIV testing<br>• Distribution of condoms
**One Month Visit (Week 4-5)**	Research Assistant	• One month quantitative survey<br>• One month qualitative survey (~5%)<br>• Screening for adverse events
**Intervention Visit 3 (Week 6-7)**	Peer Educator	• Screening for adverse events<br>• Discussion of any difficulty with HIV self test use (in HIVST arms)<br>• Referral to care and standard HIV testing<br>• Distribution of condoms

For peer review only - http://bmjopen.bmj.com/site/about/guidelines.xhtml

**Intervention Visit 4 (Week 10-12)**	Peer Educator	• Screening for adverse events<br>• Discussion of any difficulty with HIV self test use (in HIVST arms)<br>• Referral to care and standard HIV testing<br>• Distribution of condoms<br>• Distribution of second HIV self-test or coupon (in HIVST arms)
**Four Month Visit (Week 16-18)**	Research Assistant	• Four month quantitative survey<br>• Four month qualitative survey (~5%)<br>• Screening for adverse events<br>• HIV status assessment

For peer review only

Table 3. Summary of study endpoints

Endpoint	Operationalization
Primary Endpoint	
Tested for HIV in the past month	▪ Recent HIV testing measured by asking participants when they last tested and where (in all arms of the study)
Secondary Endpoints	
Use of HIV self-test	▪ Measured by buying back unused HIV self-tests at four-month visit
Awareness of HIV status	▪ Measured using a three-step approach: 1) asking participants if they know what their status is2) offering participants a small financial gift if they can correctly tell the interviewer what their HIV status is; 3) confirming HIV status with a rapid test.
Linkage to care and confirmatory testing	▪ Measured in two ways: 1) during follow-up visits, asking participants if they received confirmatory testing and linked to care and 2) collection of referral cards linking individual HIV self-tests to individuals via a unique identification number
Sexual behaviors	▪ Measured via computer-assisted personal interview (CAPI), including number of sexual behaviors, event-level sexual behavior data, and condom use with primary and casual partners<br>▪ Qualitatively assessed to understand sexual behaviors in relation to HIV testing, particularly within couples
HIV testing norms and stigma related to HIV testing	▪ Qualitatively assessed to understanding perceptions of HIV related stigma, barriers and facilitators to HIV testing, normative beliefs related to HIV testing
Cost-effectiveness	▪ Costing data for project implementation will be collected throughout the course of the intervention
Safety Endpoints	
Misuse of HIV self-tests	▪ Including difficulty conducting the test (i.e., mistakes in taking the test, incorrect use of components of the test), difficulty reading the test<br>▪ Identified through interview and ongoing consultation with peer educators
Intimate partner violence	▪ Measured through surveillance and interviews by VHT members<br>▪ Any intimate partner violence (including verbal, physical, or sexual) will be documented and reported
Difficulty using HIV self-tests	▪ Qualitatively explored during follow-up in depth interviews with participants<br>▪ Recorded in an ongoing basis by peer educators who assist participants with using or interpreting tests

For peer review only - http://bmjopen.bmj.com/site/about/guidelines.xhtml

FIGURE LEGENDS

Figure 1. Flow diagram of study enrollment, randomization, and intervention arms

Figure 2. Map of study sites in Zambia. The black star indicates Lusaka, the site of study coordination and headquarters. The circles indicate locations of study sites, including Kapiri (blue), Chirundu (green), and Livingstone (red).

Figure 3. SPIRIT flowchart of study assessments.

For peer review only

For peer review only - http://bmjopen.bmj.com/site/about/guidelines.xhtml

REFERENCES

1 Baral S, Beyrer C, Muessig K, *et al.* Burden of HIV among female sex workers in low-income and middle-income countries: a systematic review and meta-analysis. *The Lancet Infectious Diseases* 2012; **12**: 538–49.

2 Shannon K, Strathdee SA, Goldenberg SM, *et al.* Global epidemiology of HIV among female sex workers: Influence of structural determinants. *The Lancet* 2014; : 1–17.

3 Prüss-Ustün A, Wolf J, Driscoll T, Degenhardt L, Neira M, Calleja JMG. HIV Due to Female Sex Work: Regional and Global Estimates. *PLoS ONE* 2013; **8**: e63476–7.

4 Baral S, Logie CH, Grosso A, Wirtz AL, Beyrer C. Modified social ecological model: a tool to guide the assessment of the risks and risk contexts of HIV epidemics. *BMC Public Health* 2013; **13**: 482.

5 Bekker L-G, Johnson L, Cowan F, *et al.* Combination HIV prevention for female sex workers: what is the evidence? *The Lancet* 2015; **385**: 72–87.

6 Deering KN, Lyons T, Feng CX, *et al.* Client demands for unsafe sex: The socioeconomic risk environment for HIV among street and off-street sex workers. *JAIDS Journal of Acquired Immune Deficiency Syndromes* 2013; **63**: 522–31.

7 King EJ, Maman S, Bowling JM, Moracco KE, Dudina V. The Influence of Stigma and Discrimination on Female Sex Workers' Access to HIV Services in St. Petersburg, Russia. *AIDS Behav* 2013; **17**: 2597–603.

8 Oldenburg CE, Perez-Brumer AG, Hatzenbuehler ML, *et al.* State-level structural sexual stigma and HIV prevention in a national online sample of HIV-uninfected MSM in the United States. *AIDS* 2015; : 837–45.

9 Beyrer C, Crago A-L, Bekker L-G, *et al.* An action agenda for HIV and sex workers. *The Lancet* 2015; **385**: 287–301.

10 Bodkin K, Delahunty-Pike A, O'Shea T. Reducing stigma in healthcare and law enforcement: a novel approach to service provision for street level sex workers. *International Journal for Equity in Health* 2015; **14**: 322–7.

11 UNAIDS. 90-90-90: An ambitious treatment target to help end the AIDS epidemic. 2014.

12 Haber N, Naidu K, Pillay D, Barnighausen T. HIV system assessment with longitudinal treatment cascade in Kwazulu-Natal, South Africa. San Diego, CA, 2015.

For peer review only

For peer review only - http://bmjopen.bmj.com/site/about/guidelines.xhtml

13 Krause J, Subklew-Sehume F, Kenyon C, Colebunders R. Acceptability of HIV self-testing: a systematic literature review. *BMC Public Health* 2013; **13**: 1–1.

14 Figueroa C, Johnson C, Verster A, Baggaley R. Attitudes and Acceptability on HIV Self-testing Among Key Populations: A Literature Review. *AIDS Behav* 2015; : 1–17.

15 van Rooyen H, Tulloch O, Mukoma W, *et al.* What are the constraints and opportunities for HIVST scale-up in Africa? Evidence from Kenya, Malawi and South Africa. *J Int AIDS Soc* 2015; **18**. DOI:10.7448/IAS.18.1.19445.

16 Pant Pai N, Sharma J, Shivkumar S, *et al.* Supervised and Unsupervised Self-Testing for HIV in High- and Low-Risk Populations: A Systematic Review. *PLoS Medicine* 2013; **10**: e1001414.

17 Kinsler JJ, Wong MD, Sayles JN, Davis C, Cunningham WE. The Effect of Perceived Stigma from a Health Care Provider on Access to Care Among a Low-Income HIV-Positive Population. *AIDS Patient Care and STDs* 2007; **21**: 584–92.

18 Myers JE, El-Sadr WM, Zerbe A, Branson BM. Rapid HIV self-testing: long in coming but opportunities beckon. *AIDS* 2013; **27**: 1687–95.

19 Jamil MS, Prestage G, Fairley CK, *et al.* Rationale and design of FORTH: a randomised controlled trial assessing the effectiveness of HIV self-testing in increasing HIV testing frequency among gay and bisexual men. *BMC Infectious Diseases* 2015; : 1–10.

20 Katz D, Golden M, Hughes J, Farquhar C, Stekler J. HIV self-testing increases HIV testing frequency among high-risk men who have sex with men: a randomized controlled trial. Vancouver, 2015.

21 Marley G, Kang D, Wilson EC, *et al.* Introducing rapid oral–fluid HIV testing among high risk populations in Shandong, China: feasibility and challenges. *BMC Public Health* 2014; **14**: 422–7.

22 Funnell SC, Rogers PJ. Purposeful Program Theory: Effective Use of Theories of Change and Logic Models. San Francisco, CA: Jossey-Bass, 2011.

23 Hayes R, Ayles H, Beyers N, *et al.* HPTN 071 (PopART): Rationale and design of a cluster-randomised trial of the population impact of an HIV combination prevention intervention including universal testing and treatment – a study protocol for a cluster randomised trial. *Trials* 2014; **15**: 1–17.

24 Gual A, Segura L, Contel M, Heather N, Colom J. AUDIT-3 and AUDIT-4: Effectiveness of two short ofrms of the alcohol use disorders identification test. *Alcohol and Alcoholism* 2002; **37**: 591–6.

For peer review only

For peer review only - http://bmjopen.bmj.com/site/about/guidelines.xhtml

25 Kroenke K, Spitzer RL, Williams JB. The PHQ-9: Validity of a brief depression severity measure. *J GEN INTERN MED* 2001; **16**: 606–13.

26 Luszczynska A, Scholz U, Schwarzer R. The general self-efficacy scale: Multicultural validation studies. 2005; **139**: 439–57.

27 Beattie T, Mohan HL, Bhattacharjee P, *et al.* Community Mobilization and Empowerment of Female Sex Workers in Karnataka State, South India: Associations With HIV and Sexually Transmitted Infection Risk. *American Journal of Public Health* 2014; **104**: 1516–25.

28 Stadler J, Scorgie F, van der Straten A, Saethre E. Adherence and the Lie in a HIV Prevention Clinical Trial. *Medical Anthropology* 2015; : 1–14.

29 A short technical update on self-testing for HIV. UNAIDS, 2014.

30 Corridors of Hope Southern Africa. HIV prevention needs and opportunities in four border towns. Family Health International, 2005.

31 Family Health International. Female sex workers in Border and Transportation Routes with Trend Analysis, 2000-2006. 2006; : 1–79.

32 Onyango MA, Adu-Sarkodie Y, Agyarko-Poku T, *et al.* 'It's All About Making a Life': Poverty, HIV, Violence, and Other Vulnerabilities Faced by Young Female Sex Workers in Kumasi, Ghana. *JAIDS Journal of Acquired Immune Deficiency Syndromes* 2016; **68**: S131–7.

33 Ford K, Wirawan DN, Suastina W, Reed BD, Muliawan P. Evaluation of a peer education programme for female sex workers in Bali, Indonesia. *Int J STD AIDS* 2008; **11**: 731–3.

34 Oldenburg CE, Biello KB, Colby D, *et al.* Engagement with Peer Health Educators Is Associated with Willingness to Use Pre-Exposure Prophylaxis Among Male Sex Workers in Ho Chi Minh City, Vietnam. *AIDS Patient Care and STDs* 2014; **28**: 109–12.

35 Aung T, Paw E, Aye NM, McFarland W. Coverage of HIV Prevention Services for Female Sex Workers in Seven Cities of Myanmar. *AIDS Behav* 2013; **18**: 37–41.

36 Rachakulla HK, Kodavalla V, Rajkumar H, *et al.* Condom use and prevalence of syphilis and HIV among female sex workers in Andhra Pradesh, India – following a large-scale HIV prevention intervention. 2011; : 1–16.

37 Geibel S, King'ola N, Temmerman M, Luchters S. The impact of peer outreach on HIV knowledge and prevention behaviours of male sex workers in Mombasa, Kenya. *Sexually Transmitted Infections* 2012; **88**: 357–62.

For peer review only

For peer review only - http://bmjopen.bmj.com/site/about/guidelines.xhtml

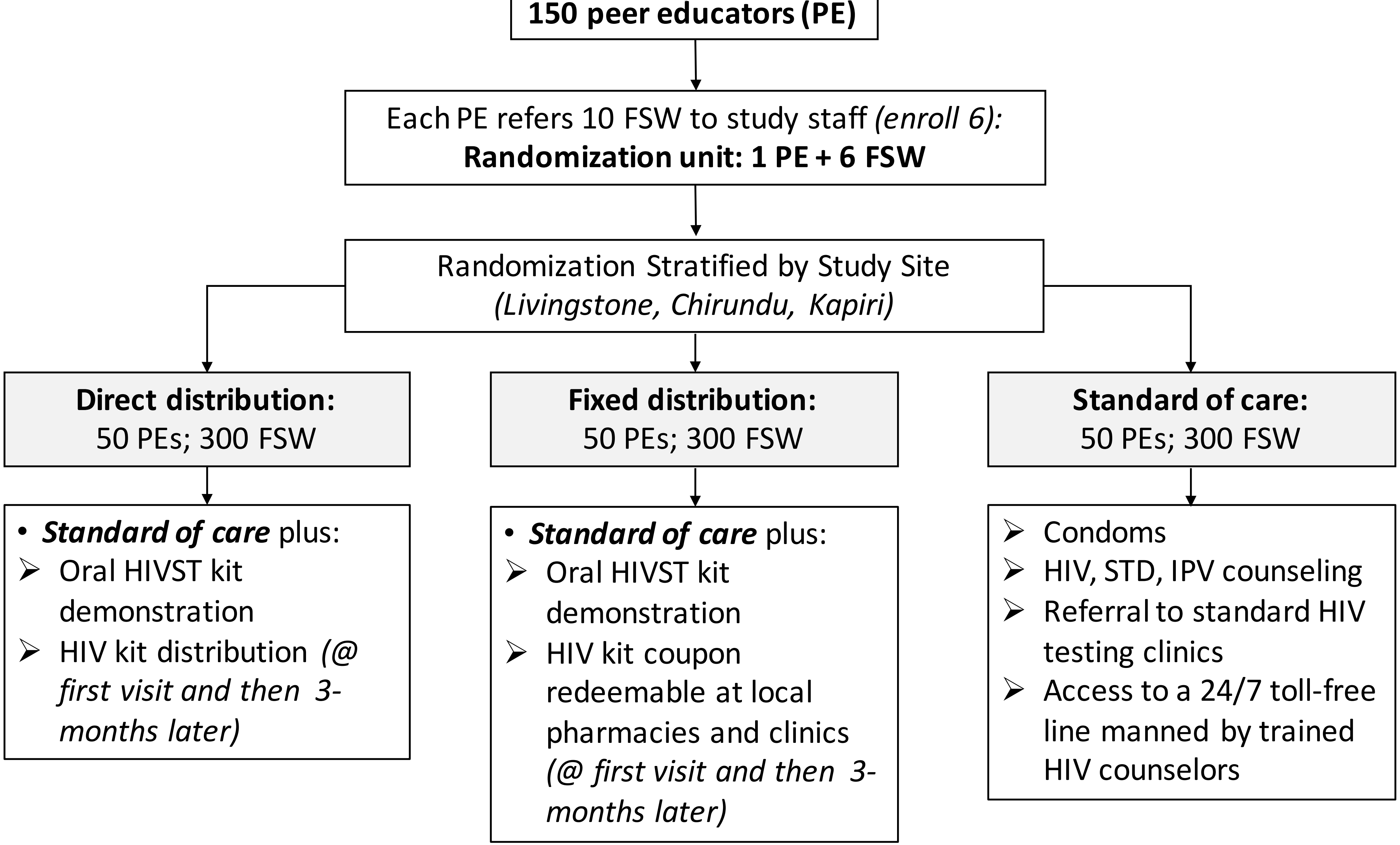
150 peer educators (PE)
Each PE refers 10 FSW to study staff (enroll 6):
Randomization unit: 1 PE + 6 FSW
Randomization Stratified by Study Site
(Livingstone, Chirundu, Kapiri)
Direct distribution:
50 PEs; 300 FSW
Fixed distribution:
50 PEs; 300 FSW
Standard of care:
50 PEs; 300 FSW
• Standard of care plus:
➢ Oral HIVST kit demonstration
➢ HIV kit distribution (@ first visit and then 3-months later)
• Standard of care plus:
➢ Oral HIVST kit demonstration
➢ HIV kit coupon redeemable at local pharmacies and clinics (@ first visit and then 3-months later)
➢ Condoms
➢ HIV, STD, IPV counseling
➢ Referral to standard HIV testing clinics
➢ Access to a 24/7 toll-free line manned by trained HIV counselors

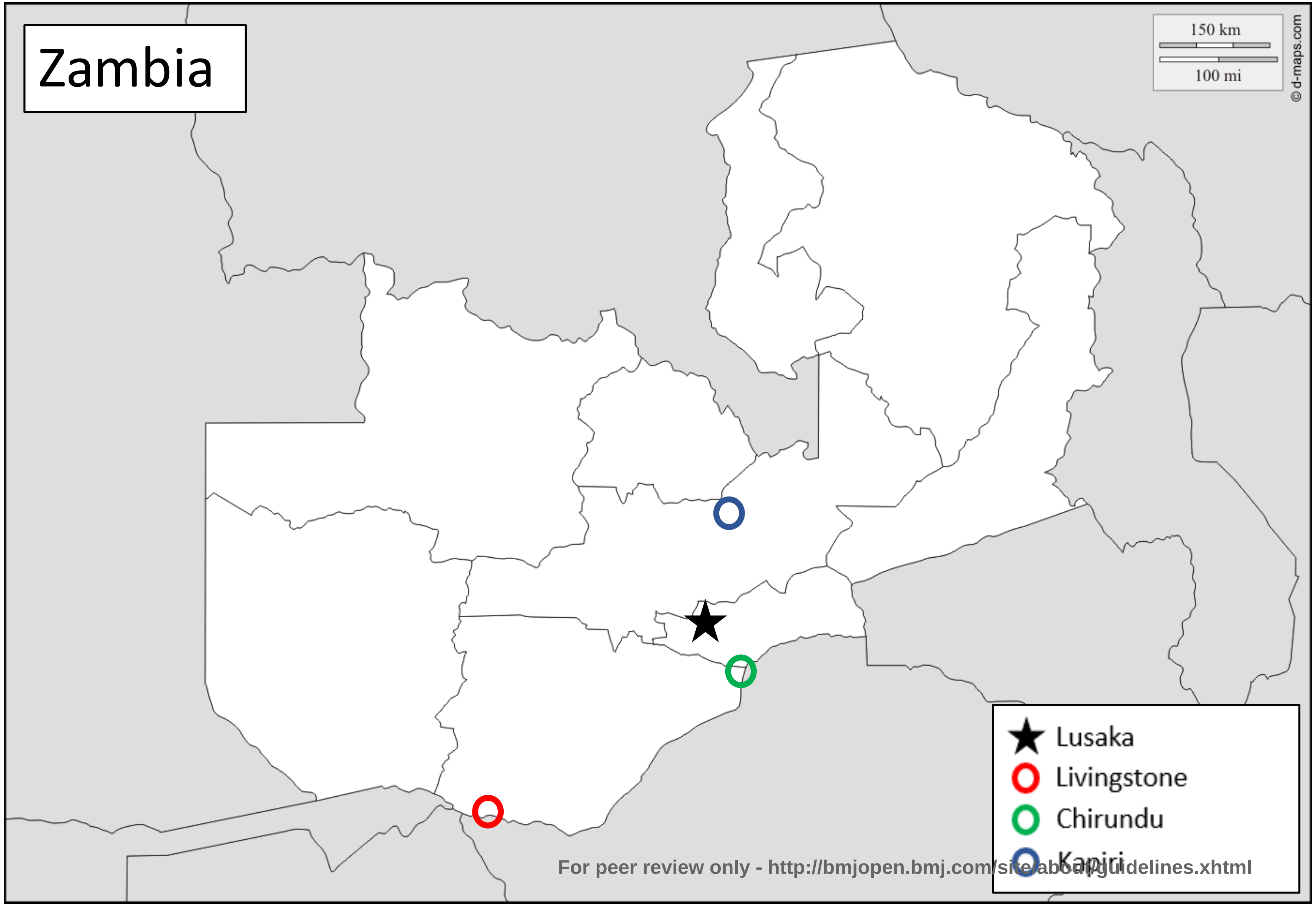

For peer review only - http://bmjopen.bmj.com/site/about/guidelines.xhtml

Figure 3. SPIRIT flowchart of study assessments

	STUDY PERIOD						
	**Enrolment**	**Allocation**	**Post-Allocation**				**Close-Out**
**TIMEPOINT****		**0**	***2 weeks***	***1 month***	***6 weeks***	***10 weeks***	***4 months***
**ENROLMENT:**							
**Eligibility screen**	X						
**Informed consent**	X						
**Allocation**		X					
**INTERVENTIONS:**							
HIVST Kit/Coupon Distribution		X				X	
Peer Educator Visit		X	X		X	X	
**ASSESSMENTS:**							
Demographics, HIV testing history, sexual behaviors, reproductive health, HIV knowledge, empowerment	X						
HIV testing history, sexual behaviors, HIV knowledge, empowerment				X			X
HIV status knowledge							X
HIV self-test kit use							X
Adverse Events			X	X	X	X	X

For peer review only

For peer review only - http://bmjopen.bmj.com/site/about/guidelines.xhtml

SPIRIT
STANDARD PROTOCOL ITEMS: RECOMMENDATIONS FOR INTERVENTIONAL TRIALS

SPIRIT 2013 Checklist: Recommended items to address in a clinical trial protocol and related documents*

Section/item	Item No	Description	Addressed on page number
**Administrative information**			
Title	1	Descriptive title identifying the study design, population, interventions, and, if applicable, trial acronym	1
Trial registration	2a	Trial identifier and registry name. If not yet registered, name of intended registry	3
	2b	All items from the World Health Organization Trial Registration Data Set	
Protocol version	3	Date and version identifier	n/a
Funding	4	Sources and types of financial, material, and other support	20
Roles and responsibilities	5a	Names, affiliations, and roles of protocol contributors	1
	5b	Name and contact information for the trial sponsor	20
	5c	Role of study sponsor and funders, if any, in study design; collection, management, analysis, and interpretation of data; writing of the report; and the decision to submit the report for publication, including whether they will have ultimate authority over any of these activities	21
	5d	Composition, roles, and responsibilities of the coordinating centre, steering committee, endpoint adjudication committee, data management team, and other individuals or groups overseeing the trial, if applicable (see Item 21a for data monitoring committee)	15

For peer review only - http://bmjopen.bmj.com/site/about/guidelines.xhtml

Introduction

Background and rationale	6a	Description of research question and justification for undertaking the trial, including summary of relevant studies (published and unpublished) examining benefits and harms for each intervention	_____5-6_______
	6b	Explanation for choice of comparators	____6_________
Objectives	7	Specific objectives or hypotheses	_____7________
Trial design	8	Description of trial design including type of trial (eg, parallel group, crossover, factorial, single group), allocation ratio, and framework (eg, superiority, equivalence, noninferiority, exploratory)	_____6________

Methods: Participants, interventions, and outcomes

Study setting	9	Description of study settings (eg, community clinic, academic hospital) and list of countries where data will be collected. Reference to where list of study sites can be obtained	______7_______
Eligibility criteria	10	Inclusion and exclusion criteria for participants. If applicable, eligibility criteria for study centres and individuals who will perform the interventions (eg, surgeons, psychotherapists)	_____8________
Interventions	11a	Interventions for each group with sufficient detail to allow replication, including how and when they will be administered	_____10-11_____
	11b	Criteria for discontinuing or modifying allocated interventions for a given trial participant (eg, drug dose change in response to harms, participant request, or improving/worsening disease)	_____n/a_______
	11c	Strategies to improve adherence to intervention protocols, and any procedures for monitoring adherence (eg, drug tablet return, laboratory tests)	______n/a______
	11d	Relevant concomitant care and interventions that are permitted or prohibited during the trial	_____n/a______
Outcomes	12	Primary, secondary, and other outcomes, including the specific measurement variable (eg, systolic blood pressure), analysis metric (eg, change from baseline, final value, time to event), method of aggregation (eg, median, proportion), and time point for each outcome. Explanation of the clinical relevance of chosen efficacy and harm outcomes is strongly recommended	___13-15_______

For peer review only - http://bmjopen.bmj.com/site/about/guidelines.xhtml

Participant timeline	13	Time schedule of enrolment, interventions (including any run-ins and washouts), assessments, and visits for participants. A schematic diagram is highly recommended (see Figure)	__11-12_______
Sample size	14	Estimated number of participants needed to achieve study objectives and how it was determined, including clinical and statistical assumptions supporting any sample size calculations	___15__________
Recruitment	15	Strategies for achieving adequate participant enrolment to reach target sample size	___15__________
**Methods: Assignment of interventions (for controlled trials)**			
Allocation:			
Sequence generation	16a	Method of generating the allocation sequence (eg, computer-generated random numbers), and list of any factors for stratification. To reduce predictability of a random sequence, details of any planned restriction (eg, blocking) should be provided in a separate document that is unavailable to those who enrol participants or assign interventions	___8__________
Allocation concealment mechanism	16b	Mechanism of implementing the allocation sequence (eg, central telephone; sequentially numbered, opaque, sealed envelopes), describing any steps to conceal the sequence until interventions are assigned	___8__________
Implementation	16c	Who will generate the allocation sequence, who will enrol participants, and who will assign participants to interventions	___8__________
Blinding (masking)	17a	Who will be blinded after assignment to interventions (eg, trial participants, care providers, outcome assessors, data analysts), and how	___n/a________
	17b	If blinded, circumstances under which unblinding is permissible, and procedure for revealing a participant's allocated intervention during the trial	__n/a_________
**Methods: Data collection, management, and analysis**			
Data collection methods	18a	Plans for assessment and collection of outcome, baseline, and other trial data, including any related processes to promote data quality (eg, duplicate measurements, training of assessors) and a description of study instruments (eg, questionnaires, laboratory tests) along with their reliability and validity, if known. Reference to where data collection forms can be found, if not in the protocol	__11___________

For peer review only - http://bmjopen.bmj.com/site/about/guidelines.xhtml

	18b	Plans to promote participant retention and complete follow-up, including list of any outcome data to be collected for participants who discontinue or deviate from intervention protocols	_n/a__________
Data management	19	Plans for data entry, coding, security, and storage, including any related processes to promote data quality (eg, double data entry; range checks for data values). Reference to where details of data management procedures can be found, if not in the protocol	__11___________
Statistical methods	20a	Statistical methods for analysing primary and secondary outcomes. Reference to where other details of the statistical analysis plan can be found, if not in the protocol	__15___________
	20b	Methods for any additional analyses (eg, subgroup and adjusted analyses)	__15 __________
	20c	Definition of analysis population relating to protocol non-adherence (eg, as randomised analysis), and any statistical methods to handle missing data (eg, multiple imputation)	__n/a__________
**Methods: Monitoring**			
Data monitoring	21a	Composition of data monitoring committee (DMC); summary of its role and reporting structure; statement of whether it is independent from the sponsor and competing interests; and reference to where further details about its charter can be found, if not in the protocol. Alternatively, an explanation of why a DMC is not needed	__7___________
	21b	Description of any interim analyses and stopping guidelines, including who will have access to these interim results and make the final decision to terminate the trial	__7___________
Harms	22	Plans for collecting, assessing, reporting, and managing solicited and spontaneously reported adverse events and other unintended effects of trial interventions or trial conduct	___15__________
Auditing	23	Frequency and procedures for auditing trial conduct, if any, and whether the process will be independent from investigators and the sponsor	__n/a_________
**Ethics and dissemination**			
Research ethics approval	24	Plans for seeking research ethics committee/institutional review board (REC/IRB) approval	___21__________

For peer review only - http://bmjopen.bmj.com/site/about/guidelines.xhtml

Protocol amendments	25	Plans for communicating important protocol modifications (eg, changes to eligibility criteria, outcomes, analyses) to relevant parties (eg, investigators, REC/IRBs, trial participants, trial registries, journals, regulators)	__n/a_________
Consent or assent	26a	Who will obtain informed consent or assent from potential trial participants or authorised surrogates, and how (see Item 32)	__21___________
	26b	Additional consent provisions for collection and use of participant data and biological specimens in ancillary studies, if applicable	__n/a__________
Confidentiality	27	How personal information about potential and enrolled participants will be collected, shared, and maintained in order to protect confidentiality before, during, and after the trial	___21_________
Declaration of interests	28	Financial and other competing interests for principal investigators for the overall trial and each study site	___21__________
Access to data	29	Statement of who will have access to the final trial dataset, and disclosure of contractual agreements that limit such access for investigators	__n/a_________
Ancillary and post-trial care	30	Provisions, if any, for ancillary and post-trial care, and for compensation to those who suffer harm from trial participation	___n/a________
Dissemination policy	31a	Plans for investigators and sponsor to communicate trial results to participants, healthcare professionals, the public, and other relevant groups (eg, via publication, reporting in results databases, or other data sharing arrangements), including any publication restrictions	__n/a_________
	31b	Authorship eligibility guidelines and any intended use of professional writers	___n/a________
	31c	Plans, if any, for granting public access to the full protocol, participant-level dataset, and statistical code	___21__________
**Appendices**			
Informed consent materials	32	Model consent form and other related documentation given to participants and authorised surrogates	__n/a_________
Biological specimens	33	Plans for collection, laboratory evaluation, and storage of biological specimens for genetic or molecular analysis in the current trial and for future use in ancillary studies, if applicable	__n/a_________

For peer review only

For peer review only - http://bmjopen.bmj.com/site/about/guidelines.xhtml

*It is strongly recommended that this checklist be read in conjunction with the SPIRIT 2013 Explanation & Elaboration for important clarification on the items. Amendments to the protocol should be tracked and dated. The SPIRIT checklist is copyrighted by the SPIRIT Group under the Creative Commons "Attribution-NonCommercial-NoDerivs 3.0 Unported" license.

For peer review only

For peer review only - http://bmjopen.bmj.com/site/about/guidelines.xhtml

BMJ Open

The Zambian Peer Educators for HIV Self-Testing (ZEST) Study: Rationale and design of a cluster randomized trial of HIV self-testing among female sex workers in Zambia

Journal:	*BMJ Open*
Manuscript ID	bmjopen-2016-014780.R1
Article Type:	Protocol
Date Submitted by the Author:	02-Jan-2017
Complete List of Authors:	Oldenburg, Catherine; University of California San Francisco, Francis I Proctor Foundation<br>Ortblad, Katrina; Harvard T.H. Chan School of Public Health, Global Health and Population<br>Chanda, Michael; John Snow, Inc<br>Mwanda, Kalasa; John Snow, Inc<br>Nicodemus, Wendy; John Snow, Inc<br>Sikaundi, Rebecca; John Snow, Inc<br>Fullem, Andrew; John Snow, Inc<br>Barresi, Leah; Harvard T.H. Chan School of Public Health, Epidemiology<br>Harling, Guy; Harvard School of Public Health, Department of Global Health and Population<br>Bärnighausen, Till; University of Heidelberg
**Primary Subject Heading**:	HIV/AIDS
Secondary Subject Heading:	Global health, Health services research
Keywords:	female sex workers, HIV testing, HIV self-testing, Sub-Saharan Africa, HIV & AIDS < INFECTIOUS DISEASES

SCHOLARONE™ Manuscripts

For peer review only

The Zambian Peer Educators for HIV Self-Testing (ZEST) Study: Rationale and design of a cluster randomized trial of HIV self-testing among female sex workers in Zambia

Catherine E. Oldenburg[1,2], Katrina F. Ortblad[3], Michael M. Chanda[4], Kalasa Mwanda[4], Wendy Nicodemus[4], Rebecca Sikaundi[4], Andrew Fullem[5], Leah G. Barresi[1], Guy Harling[3,6], Till Bärnighausen[3,7,8]

[1]Department of Epidemiology, Harvard T.H. Chan School of Public Health, Boston, MA, USA
[2]Francis I. Proctor Foundation, University of California, San Francisco, USA
[3]Department of Global Health and Population, Harvard T.H. Chan School of Public Health, Boston, MA, USA
[4]John Snow, Inc, Lusaka, Zambia
[5]John Snow, Inc, Boston, MA, USA
[6]Research Department of Infection and Population Health, University College London, UK
[7]Africa Health Research Institute, Somkhele, South Africa
[8]Institute of Public Health, Faculty of Medicine, University of Heidelberg, Heidelberg, Germany

Corresponding Author
Catherine E. Oldenburg, ScD, MPH
513 Parnassus Ave, Room S334
San Francisco, CA 94143-0412
catherine.oldenburg@ucsf.edu

Word Count: 5,092
Abstract Word Count: 345
Tables: 3
Figures: 3
References: 36

For peer review only

ABSTRACT

Background. HIV testing and knowledge of status are starting points for HIV treatment and prevention interventions. Among female sex workers (FSW), HIV testing and status knowledge remain far from universal. HIV self-testing (HIVST) is an alternative to existing testing services for FSW, but to date little evidence exists how it can be effectively and safely implemented. Here, we describe the rationale and design of a cluster randomized trial designed to inform implementation and scale-up of HIVST programs for FSW in Zambia.

Methods. The Zambian Peer Educators for HIV Self-Testing (ZEST) study is a three-arm cluster randomized trial taking place in three towns in Zambia. Participants (N=900) are eligible if they are women who have exchanged sex for money or goods in the previous one month, are HIV negative or status unknown, have not tested for HIV in the previous three months, and are at least 18 years old. Participants are recruited by peer educators working in their communities. Participants are randomized to one of three arms: 1) direct distribution (in which they receive an HIVST from the peer educator directly); 2) fixed distribution (in which they receive a coupon with which to collect the HIVST from a drug store or health post); or 3) standard of care (referral to existing HIV testing services only, without any offer of HIVST). Participants are followed at one and four months following distribution of the first HIVST. The primary endpoint is HIV testing in the past month measured at the one- and four-month visits.

Ethics and Dissemination. This study was approved by the Institutional Review Boards at Harvard T.H. Chan School of Public Health in Boston, USA and ERES Converge in

For peer review only - http://bmjopen.bmj.com/site/about/guidelines.xhtml

Lusaka, Zambia. The findings of this trial will be presented at local, regional, and international meetings and submitted to peer-reviewed journals for publication.

ClinicalTrials.gov: NCT# NCT02827240

Keywords: HIV testing; HIV self-testing; female sex workers; cluster randomized trial

Strengths and limitations of this study

- This study will be one of the first studies reporting the effectiveness and safety of an HIV self-testing intervention among female sex workers (FSW)
- The study will enroll a large sample of FSW from three towns in Zambia
- The results will allow for assessment of the feasibility and acceptability of HIV self-test kits in general
- The results will also inform the choice of health systems delivery of HIVST for FSW. In particular, it will provide evidence on whether HIVST are used by FSW if they are accessible via existing distribution points within the health system
- This study does not rely on clinic or other venue-based sampling, and thus will provide evidence on HIVST for all FSW, including those who do not routinely utilize existing services
- Limitations of this study include the self-reported nature of many of the outcomes, which may lead to social desirability biases
- To overcome social desirability biases in the reporting of outcomes, in particular the use of HIVST and HIV status knowledge, we utilize several innovative measurement approaches

For peer review only - http://bmjopen.bmj.com/site/about/guidelines.xhtml

For peer review only

For peer review only - http://bmjopen.bmj.com/site/about/guidelines.xhtml

INTRODUCTION

Female sex workers (FSW), defined as women who exchange sex for money or goods, bear a disproportionate burden of the HIV epidemic globally.[1,2] Global HIV prevalence among FSW has been estimated at nearly 12%, with HIV prevalence rising to more than 30% for FSW living in countries with medium and high background HIV epidemics.[1] In Sub-Saharan Africa in 2011, an estimated 17.8% of all infections in women were attributed to engagement in sex work.[3] Vulnerability to HIV among FSW occurs at multiple levels[4], which potentiates the HIV epidemic in this population.[2,5] Determinants of HIV among FSW occur at the individual level (behavioral and biological factors, such as sexually transmitted infections), dyad and partner level (such as power dynamics within relationships), work environment level (organizational structures where individuals work, including venue policies and availability of condoms in venues)[6], and community and macrostructural levels (including community structure and organization and legal and policy environments).[2]

In 2014, the Joint United Nations Programme on HIV and AIDS (UNAIDS) put forth a new global target for controlling and ultimately ending the HIV epidemic.[7] The 90-90-90 target is a three-part treatment target that aims to have 90% of people living with HIV aware of their status, 90% on treatment, and 90% virally suppressed by the year 2020, ultimately resulting in viral suppression for 73% of HIV-infected individuals worldwide. Such a strategy requires regular HIV testing for individuals who are at ongoing risk of HIV, including FSW. However, significant gaps remain in HIV testing coverage in Sub-Saharan Africa.[8] Given the large burden of HIV among FSW and the

For peer review only - http://bmjopen.bmj.com/site/about/guidelines.xhtml

unique barriers to HIV prevention faced by this population, evidence of strategies that are specifically designed for this population are urgently needed.

Stigma and discrimination can affect access to HIV testing among FSW.[7-10] FSW may be affected by multiple, intersecting forms of stigma. Stigma is broadly defined as negative attitudes, relative powerlessness, and loss of status related to a particular characteristic.[9] Stigma can be experienced as enacted (explicit actions such as derogatory language, active discrimination, or assault), perceived (the expectation of enacted stigma), or self-stigma (internalization of stigma).[10] For FSW, stigma can be due to engagement in sex work itself, or from HIV stigma, particularly in contexts of high HIV burden, and can come from family or other community members, partners, and healthcare providers. These experiences of stigma may be exacerbated by unequal power dynamics, poverty and financial reliance on sex work, and experiences of violence. Taken together, the multidimensionality of experiences of stigma and discrimination may greatly influence uptake of HIV testing. Interventions that mitigate stigma as a barrier to HIV testing may be especially powerful for FSW.

Oral HIV self-testing (henceforth, HIVST) is an alternative to clinic or other healthcare provider testing (e.g., home-based HIV testing and counseling) that may overcome some barriers to traditional HIV testing strategies for some populations.[11-14] HIVST has been shown to be acceptable in a variety of populations globally[11], but very little data exist on the acceptability and feasibility of HIV self-testing among FSW.[13] For FSW in particular, HIVST may be a particularly good option as it offers privacy protection, efficiency, and flexibility. FSWs are a particularly vulnerable population regarding potential harms and threats to livelihood if others are to learn they are HIV

For peer review only

positive. HIVST may offer an additional degree of privacy by allowing women to test in a place of their choosing. HIVST may be efficient for FSW. Current recommendations indicate that FSW should ideally test for HIV every three months. HIVST could reduce the burden of transport to clinics for traditional testing. Finally, HIVST is flexible. Existing clinics may not offer HIV testing services during hours which are feasible for FSW, who often sleep during the day and work during the night. HIVST can overcome each of these limitations by allowing a woman to test at a time and a place that is convenient and acceptable to her.

A rapid oral HIVST has been approved in the United States, which uses fluid from the oral mucosa and is read in 20 minutes.[15] The test, OraQuick (OraSure Technologies Inc, Bethlehem, PA, USA), has a sensitivity of 91.7% and a specificity of 99.9% among untrained users.[16] All individuals who test positive with this test are encouraged to get a blood-based confirmatory test. Among men who have sex with men (MSM) in Seattle, a randomized controlled trial demonstrated a significant increase in HIV testing coverage among individuals receiving HIVST.[17] Although HIVST may be an acceptable alternative to regular HIV testing for FSW, to date minimal evidence of the use of HIV self-testing among FSW exists.[18] The acceptability, uptake, and effectiveness and safety of HIVST among FSW remains largely unknown.

Here, we describe a cluster randomized controlled trial that addresses this critical gap in the HIVST evidence for FSW. In the trial, we test two health service delivery approaches, a direct distribution system where a FSW participant is directly given an HIVST by a peer, and a fixed distribution system where the FSW participant collects the HIVST from a fixed point such as a pharmacy or health post. The trial seeks to

For peer review only - http://bmjopen.bmj.com/site/about/guidelines.xhtml

determine the effectiveness and safety of these two HIVST delivery approaches, compared to standard of care, for improving HIV testing coverage and knowledge of HIV status.

METHODS/DESIGN

Theory of Change

The intervention tested in the ZEST study, and data collected throughout the course of the study, was guided *a priori* by a theory of change developed through mental models ad deductive development.[19] Briefly, we theorized that the distribution of HIV self-test kits via peer educators would lead to improved status knowledge by reducing barriers to HIV testing such as stigma hours of clinic operation. Enacted or perceived sex work stigma from healthcare providers and from the community may be addressed by HIVST, by allowing individuals to test for HIV in private without fear of being seen in the clinic and without fear of judgment from providers. This would lead to improved uptake of HIV testing, which would lead to improved knowledge of status and ultimately reduce time to linkage to care. However, it is also possible that a community-based intervention such as HIVST could be unsuccessful if individuals are concerned about others discovering their HIV status.

Study Design

This is a non-blinded cluster randomized trial. Individuals are enrolled as members of a peer educator group, with a target enrollment of six FSW per peer educator (with five allowed if there are recruitment difficulties). The peer educator-FSW group is

For peer review only

138 randomized in a 1:1:1 fashion to one of the three study arms: 1) peer educator distribution of HIVST directly to participants (*direct distribution*), 2) peer educator distribution of a coupon valid for an HIVST at a fixed distribution point (*fixed distribution*), or 3) *standard of care*. Participants will be followed for four months from enrollment, with follow-up assessments at Months 1 and 4 (Figure 1).

Specific Aims

The specific aims of this trial are (1) establish the effectiveness of two different distribution approaches (direct and fixed distribution) for increasing HIV testing coverage among FSW; (2) establish the effectiveness of the two distribution approaches on increasing HIV status knowledge among FSW; (3) measure HIV self-testing uptake by distribution approach; and (4) measure linkage to care by the different approaches. Additionally, the trial will measure the impact of HIVST using the two distribution approaches on a number of important safety endpoints. The trial design will allow for comparison of the two distribution approaches to each other as well as to standard HIV testing.

Study Oversight

An independent Scientific Oversight Committee (SOC) oversees the data collected as part of this study. The SOC contains members with expertise in HIV epidemiology, statistics, ethics, and female sex work. The SOC reviews any reports of serious adverse events during the course of the trial, and will convene a meeting after

For peer review only

For peer review only - http://bmjopen.bmj.com/site/about/guidelines.xhtml

approximately one-third of the subjects have reached their one-month visit. Due to the short duration of the study, there are no formal statistical stopping rules.

Setting

This study is taking place in three border and transit towns in Zambia: Chirundu, Livingstone, and Kapiri (Figure 2). Chirundu and Livingstone are located on the Zambia-Zimbabwe border, and are a major transportation points for people and goods. Kapiri is north of the capital, Lusaka, and is a transit hub, with a large weigh station where many truckers stop for the night or longer. Study headquarters and coordination for the three sites is located in the capital, Lusaka.

Recruitment

Participants are recruited by peer educators. The peer educators are current or former FSW themselves who were recruited by study staff and who have undergone a two-day training in study procedures and HIV self-testing. Peer educators, who are not considered participants in this study, were recruited from current and former sex work organizations working in each of the study towns. Interested potential participants are referred to study staff after the peer educator describes the study to them. A research assistant then conducts a phone screening to determine whether or not the participant is eligible. Formal eligibility assessments are then done in person with the research assistant and eligible individuals complete an informed consent procedure. Written informed consent is obtained from all participants.

For peer review only

For peer review only - http://bmjopen.bmj.com/site/about/guidelines.xhtml

Inclusion and Exclusion Criteria

Complete inclusion and exclusion criteria are summarized in Table 1. Participants must be women 18 years of age or older who self-report exchanging sex (including vaginal, anal, and/or oral) for money or goods in the last month.

Individuals who are currently residing in the PopART study catchment area[20] in Livingstone will be excluded from this study, in addition to any individual who reports concomitant enrollment in another HIV prevention study. The PopART study includes a household HIV testing program, which would bias outcomes in this study if individuals were being regularly tested as part of another HIV prevention study.

Randomization

Peer educator groups (the 5-6 women recruited by a particular peer educator and enrolled in the study) are randomized as a unit. Group randomization occurs after all members of the group have been enrolled in the study and completed their baseline questionnaire. Groups are randomized to one of the three study arms using a computer generated randomization list in random blocks of 3, 6, and 9 and stratified by study site (Livingstone, Chirundu, and Kapiri). Randomized study arm assignments are placed in an opaque envelope, and once all participants in a peer educator group have been enrolled, the research assistant obtains the envelope from the research coordinator or the site coordinator and opens the envelope with the peer educator.

Study Procedures

For peer review only

For peer review only - http://bmjopen.bmj.com/site/about/guidelines.xhtml

An overview of the study procedures and activities is summarized in Table 2 and Figure 3.

Peer Educator Visits

In all study arms, peer educators will meet with participants at least four times (approximately monthly) over the course of the study. The first peer educator intervention visit (Intervention Visit 1) will be a group-based visit, and includes simple HIV prevention information, information on where to go for HIV testing, and condom provision. The initial group-based intervention is organized by the peer educator with assistance from the research assistant, and occurs in a private location of mutual convenience for the group members (such as study office, or near a work place). In the HIVST arms, peer educators will provide a brief overview of how to use the self-test kits, who to contact if participants have problems or concerns, and the importance of as well as how to obtain additional care, including confirmatory testing and linkage to care. Follow-up visits (Intervention Visits 2-4) with peer educators are comprised of brief one-on-one check-in visits, during which peer educators will distribute condoms, discuss any issues participants have with the HIVST kits or other elements of the study, and screen for adverse events including intimate partner violence. Between visits, peer educators will be available should participants need help with any issue relating to safe sex, HIV, violence, or any other elements of the study. Peer educators do not specifically discuss HIV status with any participant unless the participant seeks out the peer educator for advice. Participants are given alternatives should they need help with HIVST or another element of the study but do not want to disclose their HIV status to the peer educator. In

For peer review only

For peer review only - http://bmjopen.bmj.com/site/about/guidelines.xhtml

particular, participants in all study arms also have access to an anonymous 24-hour hotline provided via the study. Participants will be instructed by the research assistant that they can call the 24-hour hotline at any time if they have difficulty with using the HIV self-test kit, need counseling related to HIV testing or gender-based violence, or need any other form of assistance over the duration of the study.

Direct Distribution Arm

In the direct distribution arm, peer educators will directly distribute HIVST kits to participants during the first intervention visit. The HIVST kit consists of a single OraQuick in-home HIV self-test and instructions translated into English, Nyanja, and Bemba. The instructions are a step-by-step pictorial and written guide for using the test and interpreting the results.

Peer educators offer a second HIVST kit to participants in the direct distribution arm at the fourth intervention visit. There is no HIV status requirement for distribution of a second test. The second test is distributed at the last peer educator visit, three months after the first, to minimize contamination of study arms.

Fixed Distribution Arm

In the fixed distribution arm, peer educators give participants a coupon which they can exchange for an HIVST kit at a participating fixed distribution facility. These facilities include drug stores and health posts that have agreed to participate in the study and have received basic training on HIVST. Staff at the fixed distribution points collect the coupons when they are exchanged for an HIVST kit and give them to research staff

For peer review only

For peer review only - http://bmjopen.bmj.com/site/about/guidelines.xhtml

on a weekly basis. These coupons include a bar code that is scanned by study staff to record which participants collected the HIVST kit.

Peer educators offer a second coupon to participants at the fourth intervention visit to individuals, similar to the direct distribution arm, that can be used to obtain the second HIVST kit.

Standard of Care Arm

In the standard of care arm, peer educators give participants information about existing HIV testing services where participants can go for testing. In Livingstone, Chirundu, and Kapiri, available options generally include government facilities, non-profit faith-based facilities, and private facilities. While clinics designed specifically for female sex workers were previously in operation in these cities, funding constraints forced these facilities to close in 2015. The information that participants receive about HIV testing facilities is customized to the city of their recruitment, and identical information is given to participants in each of the three arms.

In all study arms, participants will receive a linkage to care card from their peer educator that they will be requested to bring if they seek confirmatory testing and HIV care.

Data Collection

Baseline Survey: Enrollment

At the enrollment visit, all participants complete a quantitative questionnaire administered by a research assistant. Quantitative data are collected directly on a tablet.

For peer review only

For peer review only - http://bmjopen.bmj.com/site/about/guidelines.xhtml

All assessments are conducted in a private room at a time and location that is convenient for the participant. The baseline survey covers broad topics including demographics and household composition, sex work and professional history, reproductive health and contraception, sexual behaviors, HIV testing history, attitudes towards HIV self-testing, access to healthcare, and measures of substance use[21], depression[22], social support, self-efficacy[23], and empowerment.[24]

<u>One-Month and Four-Month Visits</u>

Follow-up surveys will be conducted at the one month and four month visits from Intervention Visit 1 (see Table 2). As with the baseline survey, all quantitative data will be collected via tablet, and qualitative data (Four Month Visit only) are collected via audio recorder. Participants in all arms of the study will be asked about their recent HIV testing history, sexual behaviors, access to healthcare, and linkage to care. In the HIVST arms of the study, participants will additionally be asked about their experiences with the HIVST kit, including if they used it and details about their experience using the test, as well as their experience with the peer educator. Participants in all arms are asked again at one and four months about HIVST acceptability and perceived likelihood of using such tests in the future.

<u>Qualitative Interviews</u>

Approximately 5% of the sample (15 participants per arm, 45 in total) are randomly selected at baseline to participate in individual qualitative in-depth interviews. During baseline screening, a random subset of participants will be asked if they would be

For peer review only - http://bmjopen.bmj.com/site/about/guidelines.xhtml

interested in participating in the qualitative phase of the study. Individual in-depth interviews are conducted by trained research assistants at Baseline and at the Four-Month follow-up visit (Table 2). All qualitative data are collected via audio recorder. Baseline interviews explore community norms related to HIV testing, multilevel (including individual, structural, and interpersonal) barriers to accessing HIV testing and other healthcare, experiences in sex work, and baseline perceptions of HIV self-testing. At follow-up, qualitative interviews focus on experiences using the HIV self-test for individuals who are in one of the two intervention arms, and individuals in all arms will be asked about their perceptions of the HIV self-test and how HIVST might be implemented at scale. In all arms at follow-up, the interviews will explore empowerment and self-efficacy related to HIV testing and HIV status disclosure.

Endpoint Measurement

Study effectiveness and safety endpoints are summarized in Table 3. The primary effectiveness endpoint for the study is the proportion of participants who tested in the previous month as assessed at the one-month follow-up visit. Secondary effectiveness endpoints include testing at four months, awareness of HIV status, HIVST kit use (restricted to the HIVST arms), linkage to care, and adverse events. Safety endpoints include

<u>Recent HIV Testing</u>

For peer review only

Recent HIV testing is assessed by self-report at the one-month and four-month visits. Participants are asked when their last HIV test was, and then asked where their test was to determine if their last test was an HIVST or other.

HIV Status Knowledge

Correct reporting of HIV status via self-report is subject to social desirability bias. To accurately measure whether an individual knows their HIV status, they must both correctly report knowledge of their HIV status and be willing to take an HIV test. To overcome some of the bias in ascertaining self-reported HIV status, we use a three-step approach. First, participants are asked if they know their current HIV status. They are then asked to tell the research assistant their current HIV status, and are told to take their best guess if they do not know. Participants are then asked to take a rapid HIV test, and are counseled that this test is entirely voluntary. To reduce bias in self-reporting HIV status, participants are told that they will receive a small gift (worth approximately USD$1) if they correctly report their status. However, after the assessment, all participants will receive the gift regardless of their reported status, thus the cash transfer is perceived as conditional but is actually unconditional for ethical reasons. Pre- and post-test counseling will be available to all individuals who participate in the HIV status assessment. This assessment is performed only at four months.

HIVST Kit Use

Actual use of the HIVST kit may also be subject to social desirability bias. Participants may want to report that they used the HIVST kit when they did not for many

For peer review only

For peer review only - http://bmjopen.bmj.com/site/about/guidelines.xhtml

reasons, including desire to please the researchers, a sense of obligation, or misperception about what it means to use the test.[25] As an attempt to reduce bias in ascertainment of actual use of the HIVST kits, research assistants will ask participants to buy back any unused HIVST kits at the end of the study with the justification that the study has now reached completion. Participants will be offered approximately USD$1 in exchange for any unused test kits in their original packaging. Prior to this offer, participants are not told that researchers will buy back tests at the end of the study (this information is also not included in the informed consent). To avoid gossip among study participants, the first buy-back will not happen until at least one month after the last test kit has been distributed (with the assumption that all participants would have used the test kit at that point if they were planning to).

For peer review only

Stigma and Empowerment

Stigma and empowerment are measured both quantitatively and qualitatively at baseline and four months. Empowerment is measured additionally at one month. Quantitatively, sex work stigma is measured via a 6-item scale that covers enacted, perceived, and self-stigma[26], and HIV stigma is measured by a 9-item scale measuring attitudes towards individuals living with HIV.[27] Qualitatively, stigma measurements include questions related to barriers to accessing care, interactions with police, and experiences working as a sex worker. Quantitatively, empowerment is measured with a 5-item scale addressing sex worker agency and power within.[24] Qualitatively, empowerment is measured as ability to get things the participant needs, to change her

For peer review only - http://bmjopen.bmj.com/site/about/guidelines.xhtml

situation, and control over elements such as condom use. We hypothesize that access to HIVST will lead to an improved sense of empowerment.

Adverse Events

Adverse events are monitored and documented throughout the study. The primary adverse events of concern are psychological harm as a result of use of the HIV self-test (for example, if participants test positive for HIV and are not prepared for the results). Although UNAIDS has recently indicated no evidence of serious adverse events related to HIV self-testing (i.e., self-harm, violence, or human rights violations)[28], occurrence of any such events are reported to all principal investigators and the Scientific Oversight Committee (SOC) within 24 hours of study staff becoming aware of them. Other adverse events include coercive testing or unintentional or unauthorized disclosure of HIV status, which are similarly reported within 24 hours.

Statistical Analysis

The primary analysis will be intention-to-treat (ITT). The primary analysis for the quantitative data will involve a mixed-effects multilevel logistic regression model to account for clustering by peer educator group and study site (participants nested in peer educators nested in study sites). These models will include an indicator term for study arm (fixed distribution, direct distribution, or control). Outcomes, including past-month HIV testing and correct knowledge of status at four months, will be modeled as dichotomous variables.

For peer review only - http://bmjopen.bmj.com/site/about/guidelines.xhtml

Qualitative Data Analysis

Qualitative data will be analyzed using an iterative descriptive approach to characterize and describe the data at their natural level. After all interviews are transcribed and translated, a codebook will be finalized and transcripts will be coded in Dedoose, a cloud-based platform for mixed methods research.

Sample Size Considerations

The sample size calculation was based on the primary endpoint, testing for HIV in the past month assessed at the one-month visit, and the secondary endpoint, proportion of participants who correctly know their status at the four month visit. Power calculations were performed using methods for cluster randomized trials, with clustering by peer educator identity. We assume that 50% of participants will have tested in the past month in the control arm based on previous estimates of HIV testing behavior among FSW in Livingstone and Chirundu.[29,30] We estimate that in the control group, approximately 70% of participants will correctly identify their HIV status, assuming that the majority of those whose status is unknown will report that they are negative, but that approximately half of these individuals will actually be positive. Assuming 20% loss to follow-up and with 50 peer educators (clusters) per arm, a sample size of 300 participants per arm (900 total) will yield 89% power to detect a risk ratio of 1.30 for recent testing and 94% power to detect a risk ratio of 1.20 for correct status knowledge, assuming a type I error probability of 0.05 and an intracluster correlation (ICC) of 0.03.

Confidentiality

For peer review only

To protect participants' confidentiality, no identifying information will be collected during any quantitative or qualitative assessment and all collected data are de-identified. A unique study identification number will be used to link data from an individual participant. Participant identity codes are kept completely separately from electronic data, in a locked cabinet in study offices. The code will be destroyed upon completion of the study.

Data Management

Quantitative data are collected directly on a study tablet via CommCare (Dimagi, Cambridge, MA), a HIPAA-compliant, cloud-based platform for electronic data collection. Qualitative data are collected via audio recorder, which are transcribed and translated verbatim. Qualitative audio recordings are destroyed upon completion of transcription and translation.

Dissemination Plan

The results of this study will be disseminated to community, policy, and scientific stakeholders. A results community meeting will be planned upon completion of the study at each study site, during which the results of the study will be announced. We will also hold a meeting with the Ministry of Health in Lusaka to disseminate our findings, and submit a report detailing the findings of the study to inform policy. Finally, results will be shared with the scientific community via presentation at international conferences and journal publication.

For peer review only

For peer review only - http://bmjopen.bmj.com/site/about/guidelines.xhtml

DISCUSSION

Despite the high risk and large burden of HIV among FSW, HIV testing and HIV status knowledge in this population remain far from universal. A number of complex barriers at multiple levels can limit access to HIV testing for FSW, including fear of stigma and abuse, fear of loss of livelihood, and the financial and time costs of utilizing traditional HIV testing services. HIVST is a promising alternative HIV testing option for FSW, because it allows for a high degree of privacy, efficiency, and flexibility in HIV testing. The overarching goal of the ZEST trial is to provide robust evidence for the development of policy for implementation and scale-up of HIVST in Zambia with a specific focus on ensuring this technology is accessible to FSW. Working in concert with the Ministry of Health in Zambia and the World Health Organization, this trial was specifically designed to fill a crucial gap in the evidence by answering two questions: 1) is HIVST an acceptable alternative to standard HIV testing for FSW, and 2) will FSW access HIVST.

Question One is addressed through the direct distribution arm. In this arm, we will be able to assess whether individuals directly given a test actually use the test. By directly giving individuals the test, we are assessing best-case scenario uptake, where there are as few barriers as possible for individuals to access the test kit. The uptake results from this arm of the study will give robust evidence on whether HIVST is an acceptable alternative to standard and currently-available HIV testing services. Coupled with qualitative data, we will be able to measure both the uptake of HIVST, but also participant attitudes towards HIVST. This will lead to an in-depth understanding of why

For peer review only

participants did or did not use the test, and how participants view the role of HIVST for HIV testing in the future.

Although uptake and acceptability of HIVST is an important piece of evidence for HIVST policy, the design of this study allows for measurement of a more realistic scenario, in which participants must go to a physical distribution point to collect their kit. Question Two is thus addressed through the fixed distribution arm. This arm will measure the degree to which barriers to acquiring the HIVST kit in the store affect acquisition and use of the test. This will be tested by implementing the project within existing drug stores and health posts, without changing the hours or geographical locations of existing services. The direct and fixed distribution arms will therefore give two complementary pieces of information – first, are participants using the test kits, and second, are they able to access them independently.

A major strength of this study is that it does not rely on clinic-based sampling or recruitment. A potential benefit of HIVST over existing HIV testing services is that it may allow individuals who are not currently engaged in care or who have never sought HIV testing because of barriers to accessing care to test for HIV. HIVST may therefore be a gateway to care for individuals who have previously been difficult to engage in care. By working through peer educators, rather than in clinics, we will access a different subset of the population. Peer educator programs have been used in a variety of settings with different sex worker populations to increase HIV prevention knowledge.[31-36] In many cases, peer educators have unique access to and rapport with FSW. This study therefore is expected to enroll a diverse population of FSW that is likely more

For peer review only

476 generalizable than a clinic-based sample, while simultaneously allowing access to
477 populations that may not currently access care.

478 A major concern with HIVST is the potential for intimate partner violence, for
479 example if sexual partners find out about HIV status through the test. All participants will
480 be screened for intimate partner violence at baseline, one, and four months. Intimate
481 partner violence is very common in the FSW community, and those who have
482 experienced intimate partner violence will be eligible to participate, although the risks of
483 participating are explained fully. Furthermore, during follow-up peer education visits,
484 peer educators will screen for intimate partner violence, and they will also be available
485 between visits should any issues come up. Finally, a 24-hour telephone line will be
486 available to all participants for any issues, including intimate partner violence, and
487 referral will be made to counseling if desired. All reports of intimate partner violence
488 will be reviewed to determine if they are related to HIVST or trial participation. In this
489 study, we do not offer HIVST for FSW to test with their clients. While it is possible that
490 this could be a benefit for some participants, it could also put some at increased risk of
491 violence. We will collect detailed quantitative and qualitative data about with whom
492 participants used the test, if anyone. The results of this study are expected to yield
493 evidence of the impact of HIVST on safety endpoints, in particular intimate partner
494 violence.

495 Another safety concern with HIVST is the potential for depression, anxiety, or
496 suicidality as a result of learning one's HIV status. Structured interviews will contain
497 screening questions for depression, and peer educators will perform a brief screening on
498 depression, anxiety, and self-harm at each visit. Finally, participants will be instructed to

For peer review only

call the hotline should they need to discuss their HIV result with anyone or receive counseling.

In conclusion, the results of this study are expected to inform policy aimed at implementation and scale-up of HIVST for FSW in Zambia. This study will begin to address critical gaps in the literature describing evidence of HIV testing interventions for FSW in Southern Africa. We expect the results of this trial will have policy impact resulting in increased availability of HIVST in Zambia.

For peer review only

For peer review only - http://bmjopen.bmj.com/site/about/guidelines.xhtml

DECLARATIONS

Ethical Approval

Ethical approval was obtained for the Zambian Peer Educators for HIV Self-Testing Study from the Harvard T.H. Chan School of Public Health Institutional Review Board in Boston, Massachusetts and the ERES Converge Institutional Review Board in Lusaka, Zambia. Written informed consent is obtained from all participants by research assistants and study coordinators at each study site.

Availability of Data and Material

Upon completion of the trial, de-identified data will be available through the International Initiative for Impact Evaluation (3ie)'s public data access repository on Dataverse.

Competing Interests

None to report.

Funding

The Zambian Peer Educators for HIV Self-Testing Study is funded by the International Initiative for Impact Evaluation (3ie). CEO is supported in part by the National Institute on Drug Abuse T32-DA013911 and the National Institute of Mental Health R25-MH083620. KFO is supported in part by the National Institute of Allergy and Infectious Disease T32-AI007535. TB is supported in part by the National Institute of Child Health and Human Development R01-HD084233 and the National Institute of Allergy and Infectious Diseases R01-AI124389 and R01-AI112339. The study sponsor is not

For peer review only

involved in study design, collection, management, analysis or interpretation of the data, of writing reports or in the decision to submit the report for publication.

Authors' Contributions

Conceived and designed study: CEO, KFO, MMC, KM, WN, RS, AF, LGB, GH, TB. Created tables/figures: CEO, KFO, LGB. Wrote first draft of manuscript: CEO, KFO, MMC. Read and critically revised manuscript: CEO, KFO, MMC, KM, WN, RS, AF, LGB, GH, TB.

Acknowledgements

We gratefully acknowledge feedback on and conversations related to our study design from Anna Heard, Annette Brown, and Nancy Diaz.

For peer review only

Table 1. Study inclusion/exclusion criteria

Inclusion criteria	*Exclusion criteria*
• 18 years or older at enrollment	• Less than 18 years at enrollment
• Reports exchanging sex (vaginal, oral, and/or anal) for money or goods at least once in the past month	• Has not exchanged any form of sex in the past month
• Self-reported HIV negative and no recent HIV test (<3 months) OR HIV status unknown	• Self-reported to be living with HIV<br>• Self-reported HIV negative but tested in the within the last 3 months
• Permeant residence in the study town of enrollment (Livingstone, Chirundu, Kapiri)	• Planning to move out of the geographical area within 4 months<br>• Living in the PopART catchment area (Livingstone only)
• Willing to participate in peer education sessions and study assessments over 4-month study period	• Meets inclusion criteria but does not wish to participate
	• Concurrently participating in another HIV prevention study

For peer review only - http://bmjopen.bmj.com/site/about/guidelines.xhtml

Table 2. Outline of study visits

Study Time Point	*Visit By*	*Activities*
**Recruitment**	Peer Educator	• Peer educator discusses study with potentially eligible participant and refers them to study staff
**Enrollment**	Research Assistant	• Assessment of eligibility<br>• Informed consent (if eligible)<br>• Baseline quantitative survey<br>• Baseline qualitative survey (~5%)
**Randomization**	Research Assistant	• Randomization of peer educator and their cohort of participants once all group members are enrolled
**Intervention Visit 1 (Week 0)**	Peer Educator	• Group-based intervention<br>• HIV prevention counseling and distribution of condoms<br>• Training on HIV self-test use (in HIVST arms)<br>• Distribution of HIV self-tests or coupons (in HIVST arms)
**Intervention Visit 2 (Week 2-3)**	Peer Educator	• Screening for adverse events<br>• Discussion of any difficulty with HIV self test use (in HIVST arms)<br>• Referral to care and standard HIV testing<br>• Distribution of condoms
**One Month Visit (Week 4-5)**	Research Assistant	• One month quantitative survey<br>• One month qualitative survey (~5%)<br>• Screening for adverse events
**Intervention Visit 3 (Week 6-7)**	Peer Educator	• Screening for adverse events<br>• Discussion of any difficulty with HIV self test use (in HIVST arms)<br>• Referral to care and standard HIV testing<br>• Distribution of condoms

For peer review only - http://bmjopen.bmj.com/site/about/guidelines.xhtml

**Intervention Visit 4 (Week 10-12)**	Peer Educator	• Screening for adverse events<br>• Discussion of any difficulty with HIV self test use (in HIVST arms)<br>• Referral to care and standard HIV testing<br>• Distribution of condoms<br>• Distribution of second HIV self-test or coupon (in HIVST arms)
**Four Month Visit (Week 16-18)**	Research Assistant	• Four month quantitative survey<br>• Four month qualitative survey (~5%)<br>• Screening for adverse events<br>• HIV status assessment

For peer review only

Table 3. Summary of study endpoints

Endpoint	Operationalization
	Primary Effectiveness Endpoint
Tested for HIV in the past month	▪ Recent HIV testing measured by asking participants when they last tested and where (in all arms of the study)
	Secondary Effectiveness Endpoints
Use of HIV self-test	▪ Measured by buying back unused HIV self-tests at four-month visit
Awareness of HIV status	▪ Measured using a three-step approach: 1) asking participants if they know what their status is; 2) offering participants a small financial gift if they can correctly tell the interviewer what their HIV status is; 3) confirming HIV status with a rapid test.
Linkage to care and confirmatory testing	▪ Measured in two ways: 1) during follow-up visits, asking participants if they received confirmatory testing and linked to care and 2) collection of referral cards linking individual HIV self-tests to individuals via a unique identification number
Sexual behaviors	▪ Measured via computer-assisted personal interview (CAPI), including number of sexual behaviors, event-level sexual behavior data, and condom use with primary and casual partners
	Safety Endpoints
Misuse of HIV self-tests	▪ Including difficulty conducting the test (i.e., mistakes in taking the test, incorrect use of components of the test), difficulty reading the test<br>▪ Identified through interview and ongoing consultation with peer educators
Intimate partner violence	▪ Measured through surveillance and interviews by research assistants<br>▪ Any intimate partner violence (including verbal, physical, or sexual) will be documented and reported

For peer review only

FIGURE LEGENDS

Figure 1. Flow diagram of study enrollment, randomization, and intervention arms

Figure 2. Map of study sites in Zambia. The black star indicates Lusaka, the site of study coordination and headquarters. The circles indicate locations of study sites, including Kapiri (blue), Chirundu (green), and Livingstone (red).

Figure 3. SPIRIT flowchart of study assessments.

For peer review only

For peer review only - http://bmjopen.bmj.com/site/about/guidelines.xhtml

REFERENCES

1 Baral S, Beyrer C, Muessig K, *et al.* Burden of HIV among female sex workers in low-income and middle-income countries: a systematic review and meta-analysis. *The Lancet Infectious Diseases* 2012;**12**:538–49. doi:10.1016/S1473-3099(12)70066-X

2 Shannon K, Strathdee SA, Goldenberg SM, *et al.* Global epidemiology of HIV among female sex workers: influence of structural determinants. *The Lancet* 2015;**385**:55–71. doi:10.1016/S0140-6736(14)60931-4

3 Prüss-Ustün A, Wolf J, Driscoll T, *et al.* HIV Due to Female Sex Work: Regional and Global Estimates. *PLoS ONE* 2013;**8**:e63476–7. doi:10.1371/journal.pone.0063476

4 Baral S, Logie CH, Grosso A, *et al.* Modified social ecological model: a tool to guide the assessment of the risks and risk contexts of HIV epidemics. *BMC Public Health* 2013;**13**:482. doi:10.1186/1471-2458-13-482

5 Bekker L-G, Johnson L, Cowan F, *et al.* Combination HIV prevention for female sex workers: what is the evidence? *The Lancet* 2015;**385**:72–87. doi:10.1016/S0140-6736(14)60974-0

6 Deering KN, Lyons T, Feng CX, *et al.* Client demands for unsafe sex: The socioeconomic risk environment for HIV among street and off-street sex workers. *JAIDS Journal of Acquired Immune Deficiency Syndromes* 2013;**63**:522–31. doi:10.1097/QAI.0b013e3182968d39

7 UNAIDS. 90-90-90: An ambitious treatment target to help end the AIDS epidemic. 2014. http://www.unaids.org/en/resources/documents/2014/90-90-90. Accessed 19 December 2016.

8 Haber N, Naidu K, Pillay D, *et al.* HIV system assessment with longitudinal treatment cascade in Kwazulu-Natal, South Africa. In: Proceedings of the 2015 Annual Meeting of the Population Association of America; 2015 April 30-May2. San Diego, CA.

9 King EJ, Maman S, Bowling JM, *et al.* The Influence of Stigma and Discrimination on Female Sex Workers' Access to HIV Services in St. Petersburg, Russia. *AIDS Behav* 2013;**17**:2597–603. doi:10.1007/s10461-013-0447-7

10 Herek GM. Confronting sexual stigma and prejudice: Theory and practice. *Journal of Social Issues* 2007;**63**:905–25.

11 Krause J, Subklew-Sehume F, Kenyon C, *et al.* Acceptability of HIV self-testing: a systematic literature review. *BMC Public Health* 2013;**13**:735. doi:10.1186/1471-2458-13-735

12 Pant Pai N, Sharma J, Shivkumar S, *et al.* Supervised and Unsupervised Self-Testing

For peer review only - http://bmjopen.bmj.com/site/about/guidelines.xhtml

for HIV in High- and Low-Risk Populations: A Systematic Review. *PLoS Medicine* 2013;**10**:e1001414. doi:10.1371/journal.pmed.1001414.s005

13 Figueroa C, Johnson C, Verster A, *et al.* Attitudes and Acceptability on HIV Self-testing Among Key Populations: A Literature Review. *AIDS Behav* 2015;:1–17. doi:10.1007/s10461-015-1097-8

14 van Rooyen H, Tulloch O, Mukoma W, *et al.* What are the constraints and opportunities for HIVST scale-up in Africa? Evidence from Kenya, Malawi and South Africa. *J Int AIDS Soc* 2015;**18**. doi:10.7448/IAS.18.1.19445

15 Myers JE, El-Sadr WM, Zerbe A, *et al.* Rapid HIV self-testing: long in coming but opportunities beckon. *AIDS* 2013;**27**:1687–95. doi:10.1097/QAD.0b013e32835fd7a0

16 Jamil MS, Prestage G, Fairley CK, *et al.* Rationale and design of FORTH: a randomised controlled trial assessing the effectiveness of HIV self-testing in increasing HIV testing frequency among gay and bisexual men. *BMC Infectious Diseases* 2015;**15**:561. doi:10.1186/s12879-015-1300-6

17 Katz D, Golden M, Hughes J, *et al.* HIV self-testing increases HIV testing frequency among high-risk men who have sex with men: a randomized controlled trial. In: Proceedings of the 8th IAS Conference on HIV Pathogenesis Treatment and Prevention; 2015 July 18-22. Seattle, WA.

18 Marley G, Kang D, Wilson EC, *et al.* Introducing rapid oral–fluid HIV testing among high risk populations in Shandong, China: feasibility and challenges. *BMC Public Health* 2014;**14**:422–7. doi:10.1186/1471-2458-14-422

19 Funnell SC, Rogers PJ. *Purposeful Program Theory: Effective Use of Theories of Change and Logic Models*. San Francisco, CA: Jossey-Bass 2011.

20 Hayes R, Ayles H, Beyers N, *et al.* HPTN 071 (PopART): Rationale and design of a cluster-randomised trial of the population impact of an HIV combination prevention intervention including universal testing and treatment – a study protocol for a cluster randomised trial. *Trials* 2014;**15**:1–17.

21 Gual A, Segura L, Contel M, *et al.* AUDIT-3 and AUDIT-4: Effectiveness of two short ofrms of the alcohol use disorders identification test. *Alcohol and Alcoholism* 2002;**37**:591–6.

22 Kroenke K, Spitzer RL, Williams JB. The PHQ-9: Validity of a brief depression severity measure. *J GEN INTERN MED* 2001;**16**:606–13.

23 Luszczynska A, Scholz U, Schwarzer R. The General Self-Efficacy Scale: Multicultural Validation Studies. *The Journal of Psychology* 2005;**139**:439–57. doi:10.3200/JRLP.139.5.439-457

For peer review only - http://bmjopen.bmj.com/site/about/guidelines.xhtml

24 Beattie T, Mohan HL, Bhattacharjee P, *et al.* Community Mobilization and Empowerment of Female Sex Workers in Karnataka State, South India: Associations With HIV and Sexually Transmitted Infection Risk. *American Journal of Public Health* 2014;**104**:1516–25. doi:10.2105/AJPH.2014.301911)

25 Stadler J, Scorgie F, van der Straten A, *et al.* Adherence and the Lie in a HIV Prevention Clinical Trial. *Medical Anthropology* 2015;:1–14. doi:10.1080/01459740.2015.1116528

26 Oldenburg C, Biello KB, Colby D, *et al.* Stigma related to sex work among men who engage in transactional sex with men in Ho Chi Minh City, Vietnam. *Int J Public Health* 2014;**59**:833–40. doi:10.1007/s00038-014-0562-x

27 Kalichman SC, Simbayi LC, Jooste S, *et al.* Development of a Brief Scale to Measure AIDS-Related Stigma in South Africa. *AIDS Behav* 2005;**9**:135–43. doi:10.1007/s10461-005-3895-x

28 A short technical update on self-testing for HIV. UNAIDS 2014.

29 Corridors of Hope Southern Africa. HIV prevention needs and opportunities in four border towns. Family Health International 2005.

30 Family Health International. Female sex workers in Border and Transportation Routes with Trend Analysis, 2000-2009. 2009. https://www.fhi360.org/sites/default/files/media/documents/Round%204%20Behavioral%20Surveillance%20Survey%20%28BSS%29%20among%20female%20sex%20workers%20%28FSWs%29%20in%202009%20with%20trend%20data%202000-2009_0.pdf

31 Onyango MA, Adu-Sarkodie Y, Agyarko-Poku T, *et al.* “It’s All About Making a Life”: Poverty, HIV, Violence, and Other Vulnerabilities Faced by Young Female Sex Workers in Kumasi, Ghana. *JAIDS Journal of Acquired Immune Deficiency Syndromes* 2016;**68**:S131–7.

32 Ford K, Wirawan DN, Suastina W, *et al.* Evaluation of a peer education programme for female sex workers in Bali, Indonesia. *Int J STD AIDS* 2008;**11**:731–3.

33 Oldenburg CE, Biello KB, Colby D, *et al.* Engagement with Peer Health Educators Is Associated with Willingness to Use Pre-Exposure Prophylaxis Among Male Sex Workers in Ho Chi Minh City, Vietnam. *AIDS Patient Care and STDs* 2014;**28**:109–12. doi:10.1089/apc.2013.0372

34 Aung T, Paw E, Aye NM, *et al.* Coverage of HIV Prevention Services for Female Sex Workers in Seven Cities of Myanmar. *AIDS Behav* 2013;**18**:37–41. doi:10.1007/s10461-013-0523-z

35 Rachakulla HK, Kodavalla V, Rajkumar H, *et al.* Condom use and prevalence of syphilis and HIV among female sex workers in Andhra Pradesh, India – following a

For peer review only - http://bmjopen.bmj.com/site/about/guidelines.xhtml

large-scale HIV prevention intervention. *BMC Public Health* 2011;**11(Suppl 6)**: S1. doi:10.1186/1471-2458-11-S6-S1

36 Geibel S, King'ola N, Temmerman M, *et al.* The impact of peer outreach on HIV knowledge and prevention behaviours of male sex workers in Mombasa, Kenya. *Sexually Transmitted Infections* 2012;**88**:357–62. doi:10.1136/sextrans-2011-050224

For peer review only

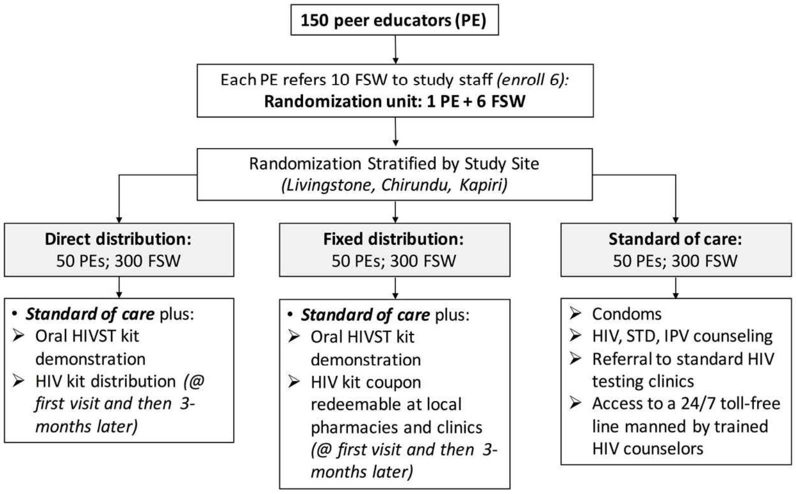

Figure 1. Flow diagram of study enrollment, randomization, and intervention arms

190x107mm (300 x 300 DPI)

For peer review only

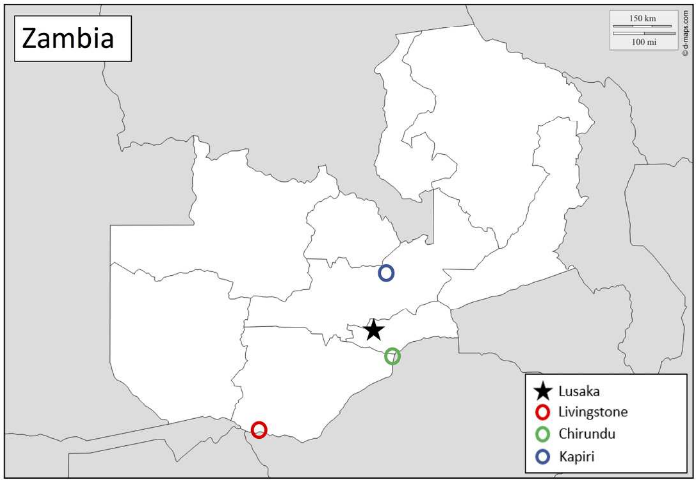

Figure 2. Map of study sites in Zambia. The black star indicates Lusaka, the site of study coordination and headquarters. The circles indicate locations of study sites, including Kapiri (blue), Chirundu (green), and Livingstone (red).

190x107mm (300 x 300 DPI)

For peer review only

Figure 3. SPIRIT flowchart of study assessments

	STUDY PERIOD						
	Enrolment	Allocation	Post-Allocation				Close-Out
**TIMEPOINT****		**0**	***2 weeks***	***1 month***	***6 weeks***	***10 weeks***	***4 months***
**ENROLMENT:**							
**Eligibility screen**	X						
**Informed consent**	X						
**Allocation**		X					
**INTERVENTIONS:**							
HIVST Kit/Coupon Distribution		X				X	
Peer Educator Visit		X	X		X	X	
**ASSESSMENTS:**							
Demographics, HIV testing history, sexual behaviors, reproductive health, HIV knowledge, empowerment	X						
HIV testing history, sexual behaviors, HIV knowledge, empowerment				X			X
HIV status knowledge							X
HIV self-test kit use							X
Adverse Events			X	X	X	X	X

Figure 3. SPIRIT flowchart of study assessments.

279x361mm (300 x 300 DPI)

SPIRIT

STANDARD PROTOCOL ITEMS: RECOMMENDATIONS FOR INTERVENTIONAL TRIALS

SPIRIT 2013 Checklist: Recommended items to address in a clinical trial protocol and related documents*

Section/item	Item No	Description	Addressed on page number
**Administrative information**			
Title	1	Descriptive title identifying the study design, population, interventions, and, if applicable, trial acronym	1
Trial registration	2a	Trial identifier and registry name. If not yet registered, name of intended registry	3
	2b	All items from the World Health Organization Trial Registration Data Set	
Protocol version	3	Date and version identifier	n/a
Funding	4	Sources and types of financial, material, and other support	26
Roles and responsibilities	5a	Names, affiliations, and roles of protocol contributors	1, 27
	5b	Name and contact information for the trial sponsor	26
	5c	Role of study sponsor and funders, if any, in study design; collection, management, analysis, and interpretation of data; writing of the report; and the decision to submit the report for publication, including whether they will have ultimate authority over any of these activities	27
	5d	Composition, roles, and responsibilities of the coordinating centre, steering committee, endpoint adjudication committee, data management team, and other individuals or groups overseeing the trial, if applicable (see Item 21a for data monitoring committee)	9

For peer review only - http://bmjopen.bmj.com/site/about/guidelines.xhtml

For peer review only

**Introduction**			
Background and rationale	6a	Description of research question and justification for undertaking the trial, including summary of relevant studies (published and unpublished) examining benefits and harms for each intervention	_____5-8_______
	6b	Explanation for choice of comparators	____7_________
Objectives	7	Specific objectives or hypotheses	_____9________
Trial design	8	Description of trial design including type of trial (eg, parallel group, crossover, factorial, single group), allocation ratio, and framework (eg, superiority, equivalence, noninferiority, exploratory)	_____7________
**Methods: Participants, interventions, and outcomes**			
Study setting	9	Description of study settings (eg, community clinic, academic hospital) and list of countries where data will be collected. Reference to where list of study sites can be obtained	______10_______
Eligibility criteria	10	Inclusion and exclusion criteria for participants. If applicable, eligibility criteria for study centres and individuals who will perform the interventions (eg, surgeons, psychotherapists)	_____11________
Interventions	11a	Interventions for each group with sufficient detail to allow replication, including how and when they will be administered	_____13-14_____
	11b	Criteria for discontinuing or modifying allocated interventions for a given trial participant (eg, drug dose change in response to harms, participant request, or improving/worsening disease)	_____n/a_______
	11c	Strategies to improve adherence to intervention protocols, and any procedures for monitoring adherence (eg, drug tablet return, laboratory tests)	______n/a______
	11d	Relevant concomitant care and interventions that are permitted or prohibited during the trial	_____n/a______
Outcomes	12	Primary, secondary, and other outcomes, including the specific measurement variable (eg, systolic blood pressure), analysis metric (eg, change from baseline, final value, time to event), method of aggregation (eg, median, proportion), and time point for each outcome. Explanation of the clinical relevance of chosen efficacy and harm outcomes is strongly recommended	___16-19_______

Participant timeline	13	Time schedule of enrolment, interventions (including any run-ins and washouts), assessments, and visits for participants. A schematic diagram is highly recommended (see Figure)	__Table 2_______
Sample size	14	Estimated number of participants needed to achieve study objectives and how it was determined, including clinical and statistical assumptions supporting any sample size calculations	___20__________
Recruitment	15	Strategies for achieving adequate participant enrolment to reach target sample size	___20__________
**Methods: Assignment of interventions (for controlled trials)**			
Allocation:			
Sequence generation	16a	Method of generating the allocation sequence (eg, computer-generated random numbers), and list of any factors for stratification. To reduce predictability of a random sequence, details of any planned restriction (eg, blocking) should be provided in a separate document that is unavailable to those who enrol participants or assign interventions	___11__________
Allocation concealment mechanism	16b	Mechanism of implementing the allocation sequence (eg, central telephone; sequentially numbered, opaque, sealed envelopes), describing any steps to conceal the sequence until interventions are assigned	___11__________
Implementation	16c	Who will generate the allocation sequence, who will enrol participants, and who will assign participants to interventions	___11__________
Blinding (masking)	17a	Who will be blinded after assignment to interventions (eg, trial participants, care providers, outcome assessors, data analysts), and how	___n/a________
	17b	If blinded, circumstances under which unblinding is permissible, and procedure for revealing a participant's allocated intervention during the trial	__n/a_________
**Methods: Data collection, management, and analysis**			
Data collection methods	18a	Plans for assessment and collection of outcome, baseline, and other trial data, including any related processes to promote data quality (eg, duplicate measurements, training of assessors) and a description of study instruments (eg, questionnaires, laboratory tests) along with their reliability and validity, if known. Reference to where data collection forms can be found, if not in the protocol	__14-16________

For peer review only - http://bmjopen.bmj.com/site/about/guidelines.xhtml

	18b	Plans to promote participant retention and complete follow-up, including list of any outcome data to be collected for participants who discontinue or deviate from intervention protocols	_n/a__________
Data management	19	Plans for data entry, coding, security, and storage, including any related processes to promote data quality (eg, double data entry; range checks for data values). Reference to where details of data management procedures can be found, if not in the protocol	__14-15________
Statistical methods	20a	Statistical methods for analysing primary and secondary outcomes. Reference to where other details of the statistical analysis plan can be found, if not in the protocol	__19___________
	20b	Methods for any additional analyses (eg, subgroup and adjusted analyses)	__19 __________
	20c	Definition of analysis population relating to protocol non-adherence (eg, as randomised analysis), and any statistical methods to handle missing data (eg, multiple imputation)	__n/a__________
**Methods: Monitoring**			
Data monitoring	21a	Composition of data monitoring committee (DMC); summary of its role and reporting structure; statement of whether it is independent from the sponsor and competing interests; and reference to where further details about its charter can be found, if not in the protocol. Alternatively, an explanation of why a DMC is not needed	__9___________
	21b	Description of any interim analyses and stopping guidelines, including who will have access to these interim results and make the final decision to terminate the trial	__10___________
Harms	22	Plans for collecting, assessing, reporting, and managing solicited and spontaneously reported adverse events and other unintended effects of trial interventions or trial conduct	___19__________
Auditing	23	Frequency and procedures for auditing trial conduct, if any, and whether the process will be independent from investigators and the sponsor	__n/a_________
**Ethics and dissemination**			
Research ethics approval	24	Plans for seeking research ethics committee/institutional review board (REC/IRB) approval	___26__________

For peer review only - http://bmjopen.bmj.com/site/about/guidelines.xhtml

Protocol amendments	25	Plans for communicating important protocol modifications (eg, changes to eligibility criteria, outcomes, analyses) to relevant parties (eg, investigators, REC/IRBs, trial participants, trial registries, journals, regulators)	__n/a_________
Consent or assent	26a	Who will obtain informed consent or assent from potential trial participants or authorised surrogates, and how (see Item 32)	__26____________
	26b	Additional consent provisions for collection and use of participant data and biological specimens in ancillary studies, if applicable	__n/a___________
Confidentiality	27	How personal information about potential and enrolled participants will be collected, shared, and maintained in order to protect confidentiality before, during, and after the trial	___21__________
Declaration of interests	28	Financial and other competing interests for principal investigators for the overall trial and each study site	___26___________
Access to data	29	Statement of who will have access to the final trial dataset, and disclosure of contractual agreements that limit such access for investigators	__26__________
Ancillary and post-trial care	30	Provisions, if any, for ancillary and post-trial care, and for compensation to those who suffer harm from trial participation	___n/a_________
Dissemination policy	31a	Plans for investigators and sponsor to communicate trial results to participants, healthcare professionals, the public, and other relevant groups (eg, via publication, reporting in results databases, or other data sharing arrangements), including any publication restrictions	__21__________
	31b	Authorship eligibility guidelines and any intended use of professional writers	___n/a_________
	31c	Plans, if any, for granting public access to the full protocol, participant-level dataset, and statistical code	___26___________
**Appendices**			
Informed consent materials	32	Model consent form and other related documentation given to participants and authorised surrogates	__n/a_________
Biological specimens	33	Plans for collection, laboratory evaluation, and storage of biological specimens for genetic or molecular analysis in the current trial and for future use in ancillary studies, if applicable	__n/a_________

For peer review only - http://bmjopen.bmj.com/site/about/guidelines.xhtml

*It is strongly recommended that this checklist be read in conjunction with the SPIRIT 2013 Explanation & Elaboration for important clarification on the items. Amendments to the protocol should be tracked and dated. The SPIRIT checklist is copyrighted by the SPIRIT Group under the Creative Commons "Attribution-NonCommercial-NoDerivs 3.0 Unported" license.

For peer review only

For peer review only - http://bmjopen.bmj.com/site/about/guidelines.xhtml

The Zambian Peer Educators for HIV Self-Testing (ZEST) Study: Rationale and design of a cluster randomized trial of HIV self-testing among female sex workers in Zambia

Journal:	*BMJ Open*
Manuscript ID	bmjopen-2016-014780.R2
Article Type:	Protocol
Date Submitted by the Author:	17-Feb-2017
Complete List of Authors:	Oldenburg, Catherine; University of California San Francisco, Francis I Proctor Foundation<br>Ortblad, Katrina; Harvard T.H. Chan School of Public Health, Global Health and Population<br>Chanda, Michael; John Snow, Inc<br>Mwanda, Kalasa; John Snow, Inc<br>Nicodemus, Wendy; John Snow, Inc<br>Sikaundi, Rebecca; John Snow, Inc<br>Fullem, Andrew; John Snow, Inc<br>Barresi, Leah; Harvard T.H. Chan School of Public Health, Epidemiology<br>Harling, Guy; Harvard School of Public Health, Department of Global Health and Population<br>Bärnighausen, Till; University of Heidelberg
**Primary Subject Heading**:	HIV/AIDS
Secondary Subject Heading:	Global health, Health services research
Keywords:	female sex workers, HIV testing, HIV self-testing, Sub-Saharan Africa, HIV & AIDS < INFECTIOUS DISEASES

SCHOLARONE™
Manuscripts

The Zambian Peer Educators for HIV Self-Testing (ZEST) Study: Rationale and design of a cluster randomized trial of HIV self-testing among female sex workers in Zambia

Catherine E. Oldenburg[1,2], Katrina F. Ortblad[3], Michael M. Chanda[4], Kalasa Mwanda[4], Wendy Nicodemus[4], Rebecca Sikaundi[4], Andrew Fullem[5], Leah G. Barresi[1], Guy Harling[3,6], Till Bärnighausen[3,7,8]

[1]Department of Epidemiology, Harvard T.H. Chan School of Public Health, Boston, MA, USA
[2]Francis I. Proctor Foundation, University of California, San Francisco, USA
[3]Department of Global Health and Population, Harvard T.H. Chan School of Public Health, Boston, MA, USA
[4]John Snow, Inc, Lusaka, Zambia
[5]John Snow, Inc, Boston, MA, USA
[6]Research Department of Infection and Population Health, University College London, UK
[7]Africa Health Research Institute, Somkhele, South Africa
[8]Institute of Public Health, Faculty of Medicine, University of Heidelberg, Heidelberg, Germany

Corresponding Author
Catherine E. Oldenburg, ScD, MPH
513 Parnassus Ave, Room S334
San Francisco, CA 94143-0412
catherine.oldenburg@ucsf.edu

Word Count: 5,145
Abstract Word Count: 345
Tables: 3
Figures: 3
References: 36

For peer review only

ABSTRACT

Background. HIV testing and knowledge of status are starting points for HIV treatment and prevention interventions. Among female sex workers (FSW), HIV testing and status knowledge remain far from universal. HIV self-testing (HIVST) is an alternative to existing testing services for FSW, but to date little evidence exists how it can be effectively and safely implemented. Here, we describe the rationale and design of a cluster randomized trial designed to inform implementation and scale-up of HIVST programs for FSW in Zambia.

Methods. The Zambian Peer Educators for HIV Self-Testing (ZEST) study is a three-arm cluster randomized trial taking place in three towns in Zambia. Participants (N=900) are eligible if they are women who have exchanged sex for money or goods in the previous one month, are HIV negative or status unknown, have not tested for HIV in the previous three months, and are at least 18 years old. Participants are recruited by peer educators working in their communities. Participants are randomized to one of three arms: 1) direct distribution (in which they receive an HIVST from the peer educator directly); 2) fixed distribution (in which they receive a coupon with which to collect the HIVST from a drug store or health post); or 3) standard of care (referral to existing HIV testing services only, without any offer of HIVST). Participants are followed at one and four months following distribution of the first HIVST. The primary endpoint is HIV testing in the past month measured at the one- and four-month visits.

Ethics and Dissemination. This study was approved by the Institutional Review Boards at Harvard T.H. Chan School of Public Health in Boston, USA and ERES Converge in

For peer review only - http://bmjopen.bmj.com/site/about/guidelines.xhtml

Lusaka, Zambia. The findings of this trial will be presented at local, regional, and international meetings and submitted to peer-reviewed journals for publication.

ClinicalTrials.gov: NCT# NCT02827240

Keywords: HIV testing; HIV self-testing; female sex workers; cluster randomized trial

Strengths and limitations of this study

- This study will be one of the first studies reporting the effectiveness and safety of an HIV self-testing intervention among female sex workers (FSW)
- The results will allow for assessment of the feasibility and acceptability of HIV self-test kits in general
- The results will provide evidence on whether HIVST is used by FSW if they it is accessible via existing distribution points within the health system
- Limitations of this study include the self-reported nature of many of the outcomes, which may lead to social desirability biases
- To overcome social desirability biases in the reporting of outcomes, in particular the use of HIVST and HIV status knowledge, we utilize several innovative measurement approaches

For peer review only

INTRODUCTION

Female sex workers (FSW), defined as women who exchange sex for money or goods, bear a disproportionate burden of the HIV epidemic globally.[1,2] Global HIV prevalence among FSW has been estimated at nearly 12%, with HIV prevalence rising to more than 30% for FSW living in countries with medium and high background HIV epidemics.[1] In Sub-Saharan Africa in 2011, an estimated 17.8% of all infections in women were attributed to engagement in sex work.[3] Vulnerability to HIV among FSW occurs at multiple levels[4], which potentiates the HIV epidemic in this population.[2,5] Determinants of HIV among FSW occur at the individual level (behavioral and biological factors, such as sexually transmitted infections), dyad and partner level (such as power dynamics within relationships), work environment level (organizational structures where individuals work, including venue policies and availability of condoms in venues)[6], and community and macrostructural levels (including community structure and organization and legal and policy environments).[2]

In 2014, the Joint United Nations Programme on HIV and AIDS (UNAIDS) put forth a new global target for controlling and ultimately ending the HIV epidemic.[7] The 90-90-90 target is a three-part treatment target that aims to have 90% of people living with HIV aware of their status, 90% on treatment, and 90% virally suppressed by the year 2020, ultimately resulting in viral suppression for 73% of HIV-infected individuals worldwide. Such a strategy requires regular HIV testing for individuals who are at ongoing risk of HIV, including FSW. However, significant gaps remain in HIV testing coverage in Sub-Saharan Africa.[8] Given the large burden of HIV among FSW and the

For peer review only - http://bmjopen.bmj.com/site/about/guidelines.xhtml

unique barriers to HIV prevention faced by this population, evidence of strategies that are specifically designed for this population are urgently needed.

Stigma and discrimination can affect access to HIV testing among FSW.[9,10] FSW may be affected by multiple, intersecting forms of stigma. Stigma is broadly defined as negative attitudes, relative powerlessness, and loss of status related to a particular characteristic.[9] Stigma can be experienced as enacted (explicit actions such as derogatory language, active discrimination, or assault), perceived (the expectation of enacted stigma), or self-stigma (internalization of stigma).[10] For FSW, stigma can be due to engagement in sex work itself, or from HIV stigma, particularly in contexts of high HIV burden, and can come from family or other community members, partners, and healthcare providers. These experiences of stigma may be exacerbated by unequal power dynamics, poverty and financial reliance on sex work, and experiences of violence. Taken together, the multidimensionality of experiences of stigma and discrimination may greatly influence uptake of HIV testing. Interventions that mitigate stigma as a barrier to HIV testing may be especially powerful for FSW.

Oral HIV self-testing (henceforth, HIVST) is an alternative to clinic or other healthcare provider testing (e.g., home-based HIV testing and counseling) that may overcome some barriers to traditional HIV testing strategies for some populations.[11-14] HIVST has been shown to be acceptable in a variety of populations globally[11], but very little data exist on the acceptability and feasibility of HIV self-testing among FSW.[13] For FSW in particular, HIVST may be a particularly good option as it offers privacy protection, efficiency, and flexibility. FSWs are a particularly vulnerable population regarding potential harms and threats to livelihood if others are to learn they are HIV

For peer review only

positive. HIVST may offer an additional degree of privacy by allowing women to test in a place of their choosing. HIVST may be efficient for FSW. Current recommendations indicate that FSW should ideally test for HIV every three months. HIVST could reduce the burden of transport to clinics for traditional testing. Finally, HIVST is flexible. Existing clinics may not offer HIV testing services during hours which are feasible for FSW, who often sleep during the day and work during the night. HIVST can overcome each of these limitations by allowing a woman to test at a time and a place that is convenient and acceptable to her.

A rapid oral HIVST has been approved in the United States, which uses fluid from the oral mucosa and is read in 20 minutes.[15] The test, OraQuick (OraSure Technologies Inc, Bethlehem, PA, USA), has a sensitivity of 91.7% and a specificity of 99.9% among untrained users.[16] All individuals who test positive with this test are encouraged to get a blood-based confirmatory test. Among men who have sex with men (MSM) in Seattle, a randomized controlled trial demonstrated a significant increase in HIV testing coverage among individuals receiving HIVST.[17] Although HIVST may be an acceptable alternative to regular HIV testing for FSW, to date minimal evidence of the use of HIV self-testing among FSW exists.[18] The acceptability, uptake, and effectiveness and safety of HIVST among FSW remains largely unknown.

Here, we describe a cluster randomized controlled trial that addresses this critical gap in the HIVST evidence for FSW. In the trial, we test two health service delivery approaches, a direct distribution system where a FSW participant is directly given an HIVST by a peer, and a fixed distribution system where the FSW participant collects the HIVST from a fixed point such as a pharmacy or health post. The trial seeks to

For peer review only - http://bmjopen.bmj.com/site/about/guidelines.xhtml

determine the effectiveness and safety of these two HIVST delivery approaches, compared to standard of care, for improving HIV testing coverage and knowledge of HIV status.

METHODS/DESIGN

Theory of Change

The intervention tested in the ZEST study, and data collected throughout the course of the study, was guided *a priori* by a theory of change developed through mental models ad deductive development.[19] Briefly, we theorized that the distribution of HIV self-test kits via peer educators would lead to improved status knowledge by reducing barriers to HIV testing such as stigma hours of clinic operation. Enacted or perceived sex work stigma from healthcare providers and from the community may be addressed by HIVST, by allowing individuals to test for HIV in private without fear of being seen in the clinic and without fear of judgment from providers. This would lead to improved uptake of HIV testing, which would lead to improved knowledge of status and ultimately reduce time to linkage to care. However, it is also possible that a community-based intervention such as HIVST could be unsuccessful if individuals are concerned about others discovering their HIV status.

Study Design

This is a non-blinded cluster randomized trial. Individuals are enrolled as members of a peer educator group, with a target enrollment of six FSW per peer educator (with five allowed if there are recruitment difficulties). The peer educator-FSW group is

For peer review only - http://bmjopen.bmj.com/site/about/guidelines.xhtml

randomized in a 1:1:1 fashion to one of the three study arms: 1) peer educator distribution of HIVST directly to participants (*direct distribution*), 2) peer educator distribution of a coupon valid for an HIVST at a fixed distribution point (*fixed distribution*), or 3) *standard of care*. Participants will be followed for four months from enrollment, with follow-up assessments at Months 1 and 4 (Figure 1).

Specific Aims

The specific aims of this trial are (1) establish the effectiveness of two different distribution approaches (direct and fixed distribution) for increasing HIV testing coverage among FSW; (2) establish the effectiveness of the two distribution approaches on increasing HIV status knowledge among FSW; (3) measure HIV self-testing uptake by distribution approach; and (4) measure linkage to care by the different approaches. Additionally, the trial will measure the impact of HIVST using the two distribution approaches on a number of important safety endpoints. The trial design will allow for comparison of the two distribution approaches to each other as well as to standard HIV testing.

Study Oversight

An independent Scientific Oversight Committee (SOC) oversees the data collected as part of this study. The SOC contains members with expertise in HIV epidemiology, statistics, ethics, and female sex work. The SOC reviews any reports of serious adverse events during the course of the trial, and will convene a meeting after

For peer review only - http://bmjopen.bmj.com/site/about/guidelines.xhtml

approximately one-third of the subjects have reached their one-month visit. Due to the short duration of the study, there are no formal statistical stopping rules.

Setting

This study is taking place in three border and transit towns in Zambia: Chirundu, Livingstone, and Kapiri (Figure 2). Chirundu and Livingstone are located on the Zambia-Zimbabwe border, and are a major transportation points for people and goods. Kapiri is north of the capital, Lusaka, and is a transit hub, with a large weigh station where many truckers stop for the night or longer. Study headquarters and coordination for the three sites is located in the capital, Lusaka.

Recruitment

Participants are recruited by peer educators. The peer educators are current or former FSW themselves who were recruited by study staff and who have undergone a two-day training in study procedures and HIV self-testing. Peer educators, who are not considered participants in this study, were recruited from current and former sex work organizations working in each of the study towns. Interested potential participants are referred to study staff after the peer educator describes the study to them. A research assistant then conducts a phone screening to determine whether or not the participant is eligible. Formal eligibility assessments are then done in person with the research assistant and eligible individuals complete an informed consent procedure. Written informed consent is obtained from all participants.

For peer review only

For peer review only - http://bmjopen.bmj.com/site/about/guidelines.xhtml

Inclusion and Exclusion Criteria

Complete inclusion and exclusion criteria are summarized in Table 1. Participants must be women 18 years of age or older who self-report exchanging sex (including vaginal, anal, and/or oral) for money or goods in the last month.

Individuals who are currently residing in the PopART study catchment area[20] in Livingstone will be excluded from this study, in addition to any individual who reports concomitant enrollment in another HIV prevention study. The PopART study includes a household HIV testing program, which would bias outcomes in this study if individuals were being regularly tested as part of another HIV prevention study.

Randomization

Peer educator groups (the 5-6 women recruited by a particular peer educator and enrolled in the study) are randomized as a unit. Group randomization occurs after all members of the group have been enrolled in the study and completed their baseline questionnaire. Groups are randomized to one of the three study arms using a computer generated randomization list in random blocks of 3, 6, and 9 and stratified by study site (Livingstone, Chirundu, and Kapiri). Randomized study arm assignments are placed in an opaque envelope, and once all participants in a peer educator group have been enrolled, the research assistant obtains the envelope from the research coordinator or the site coordinator and opens the envelope with the peer educator.

Study Procedures

For peer review only

For peer review only - http://bmjopen.bmj.com/site/about/guidelines.xhtml

An overview of the study procedures and activities is summarized in Table 2 and Figure 3.

Peer Educator Visits

In all study arms, peer educators will meet with participants at least four times (approximately monthly) over the course of the study. The first peer educator intervention visit (Intervention Visit 1) will be a group-based visit, and includes simple HIV prevention information, information on where to go for HIV testing, and condom provision. The initial group-based intervention is organized by the peer educator with assistance from the research assistant, and occurs in a private location of mutual convenience for the group members (such as study office, or near a work place). In the HIVST arms, peer educators will provide a brief overview of how to use the self-test kits, who to contact if participants have problems or concerns, and the importance of as well as how to obtain additional care, including confirmatory testing and linkage to care. Follow-up visits (Intervention Visits 2-4) with peer educators are comprised of brief one-on-one check-in visits, during which peer educators will distribute condoms, discuss any issues participants have with the HIVST kits or other elements of the study, and screen for adverse events including intimate partner violence. Between visits, peer educators will be available should participants need help with any issue relating to safe sex, HIV, violence, or any other elements of the study. Peer educators do not specifically discuss HIV status with any participant unless the participant seeks out the peer educator for advice. Participants are given alternatives should they need help with HIVST or another element of the study but do not want to disclose their HIV status to the peer educator. In

For peer review only - http://bmjopen.bmj.com/site/about/guidelines.xhtml

particular, participants in all study arms also have access to an anonymous 24-hour hotline provided via the study. Participants will be instructed by the research assistant that they can call the 24-hour hotline at any time if they have difficulty with using the HIV self-test kit, need counseling related to HIV testing or gender-based violence, or need any other form of assistance over the duration of the study.

Direct Distribution Arm

In the direct distribution arm, peer educators will directly distribute HIVST kits to participants during the first intervention visit. The HIVST kit consists of a single OraQuick in-home HIV self-test and instructions translated into English, Nyanja, and Bemba. The instructions are a step-by-step pictorial and written guide for using the test and interpreting the results.

Peer educators offer a second HIVST kit to participants in the direct distribution arm at the fourth intervention visit. There is no HIV status requirement for distribution of a second test. The second test is distributed at the last peer educator visit, three months after the first, to minimize contamination of study arms.

Fixed Distribution Arm

In the fixed distribution arm, peer educators give participants a coupon which they can exchange for an HIVST kit at a participating fixed distribution facility. These facilities include drug stores and health posts that have agreed to participate in the study and have received basic training on HIVST. Staff at the fixed distribution points collect the coupons when they are exchanged for an HIVST kit and give them to research staff

For peer review only

on a weekly basis. These coupons include a bar code that is scanned by study staff to record which participants collected the HIVST kit.

Peer educators offer a second coupon to participants at the fourth intervention visit to individuals, similar to the direct distribution arm, that can be used to obtain the second HIVST kit.

Standard of Care Arm

In the standard of care arm, peer educators give participants information about existing HIV testing services where participants can go for testing. In Livingstone, Chirundu, and Kapiri, available options generally include government facilities, non-profit faith-based facilities, and private facilities. While clinics designed specifically for female sex workers were previously in operation in these cities, funding constraints forced these facilities to close in 2015. The information that participants receive about HIV testing facilities is customized to the city of their recruitment, and identical information is given to participants in each of the three arms.

In all study arms, participants will receive a linkage to care card from their peer educator that they will be requested to bring if they seek confirmatory testing and HIV care.

Data Collection

Baseline Survey: Enrollment

At the enrollment visit, all participants complete a quantitative questionnaire administered by a research assistant. Quantitative data are collected directly on a tablet.

For peer review only - http://bmjopen.bmj.com/site/about/guidelines.xhtml

All assessments are conducted in a private room at a time and location that is convenient for the participant. The baseline survey covers broad topics including demographics and household composition, sex work and professional history, reproductive health and contraception, sexual behaviors, HIV testing history, attitudes towards HIV self-testing, access to healthcare, and measures of substance use[21], depression[22], social support, self-efficacy[23], and empowerment.[24]

<u>One-Month and Four-Month Visits</u>

Follow-up surveys will be conducted at the one month and four month visits from Intervention Visit 1 (see Table 2). As with the baseline survey, all quantitative data will be collected via tablet, and qualitative data (Four Month Visit only) are collected via audio recorder. Participants in all arms of the study will be asked about their recent HIV testing history, sexual behaviors, access to healthcare, and linkage to care. In the HIVST arms of the study, participants will additionally be asked about their experiences with the HIVST kit, including if they used it and details about their experience using the test, as well as their experience with the peer educator. Participants in all arms are asked again at one and four months about HIVST acceptability and perceived likelihood of using such tests in the future.

<u>Qualitative Interviews</u>

Approximately 5% of the sample (15 participants per arm, 45 in total) are randomly selected at baseline to participate in individual qualitative in-depth interviews. During baseline screening, a random subset of participants will be asked if they would be

For peer review only - http://bmjopen.bmj.com/site/about/guidelines.xhtml

interested in participating in the qualitative phase of the study. Individual in-depth interviews are conducted by trained research assistants at Baseline and at the Four-Month follow-up visit (Table 2). All qualitative data are collected via audio recorder. Baseline interviews explore community norms related to HIV testing, multilevel (including individual, structural, and interpersonal) barriers to accessing HIV testing and other healthcare, experiences in sex work, and baseline perceptions of HIV self-testing. At follow-up, qualitative interviews focus on experiences using the HIV self-test for individuals who are in one of the two intervention arms, and individuals in all arms will be asked about their perceptions of the HIV self-test and how HIVST might be implemented at scale. In all arms at follow-up, the interviews will explore empowerment and self-efficacy related to HIV testing and HIV status disclosure.

Endpoint Measurement

Study effectiveness and safety endpoints are summarized in Table 3. The primary effectiveness endpoint for the study is the proportion of participants who tested in the previous month as assessed at the one-month follow-up visit. Secondary effectiveness endpoints include testing at four months, awareness of HIV status, HIVST kit use (restricted to the HIVST arms), linkage to care, and adverse events. Safety endpoints include

<u>Recent HIV Testing</u>

For peer review only

Recent HIV testing is assessed by self-report at the one-month and four-month visits. Participants are asked when their last HIV test was, and then asked where their test was to determine if their last test was an HIVST or other.

HIV Status Knowledge

Correct reporting of HIV status via self-report is subject to social desirability bias. To accurately measure whether an individual knows their HIV status, they must both correctly report knowledge of their HIV status and be willing to take an HIV test. To overcome some of the bias in ascertaining self-reported HIV status, we use a three-step approach. First, participants are asked if they know their current HIV status. They are then asked to tell the research assistant their current HIV status, and are told to take their best guess if they do not know. Participants are then asked to take a rapid HIV test, and are counseled that this test is entirely voluntary. To reduce bias in self-reporting HIV status, participants are told that they will receive a small gift (worth approximately USD$1) if they correctly report their status. However, after the assessment, all participants will receive the gift regardless of their reported status, thus the cash transfer is perceived as conditional but is actually unconditional for ethical reasons. Pre- and post-test counseling will be available to all individuals who participate in the HIV status assessment. This assessment is performed only at four months.

HIVST Kit Use

Actual use of the HIVST kit may also be subject to social desirability bias. Participants may want to report that they used the HIVST kit when they did not for many

For peer review only

For peer review only - http://bmjopen.bmj.com/site/about/guidelines.xhtml

reasons, including desire to please the researchers, a sense of obligation, or misperception about what it means to use the test.[25] As an attempt to reduce bias in ascertainment of actual use of the HIVST kits, research assistants will ask participants to buy back any unused HIVST kits at the end of the study with the justification that the study has now reached completion. Participants will be offered approximately USD$1 in exchange for any unused test kits in their original packaging. Prior to this offer, participants are not told that researchers will buy back tests at the end of the study (this information is also not included in the informed consent). To avoid gossip among study participants, the first buy-back will not happen until at least one month after the last test kit has been distributed (with the assumption that all participants would have used the test kit at that point if they were planning to).

Stigma and Empowerment

Stigma and empowerment are measured both quantitatively and qualitatively at baseline and four months. Empowerment is measured additionally at one month. Quantitatively, sex work stigma is measured via a 6-item scale that covers enacted, perceived, and self-stigma[26], and HIV stigma is measured by a 9-item scale measuring attitudes towards individuals living with HIV.[27] Qualitatively, stigma measurements include questions related to barriers to accessing care, interactions with police, and experiences working as a sex worker. We hypothesize that at baseline individuals who report a greater amount of stigma will have reduced HIV testing, and that these individuals will have greater uptake of HIVST. Empowerment is defined as the process by which disenfranchised individuals acquire the ability to make choices.[24]

For peer review only - http://bmjopen.bmj.com/site/about/guidelines.xhtml

Quantitatively, empowerment is measured with a 5-item scale addressing sex worker agency and power within.[24] Qualitatively, empowerment is measured as ability to get things the participant needs, to change her situation, and control over elements such as condom use. We hypothesize that access to HIVST will lead to an improved sense of empowerment.

Adverse Events

Adverse events are monitored and documented throughout the study. The primary adverse events of concern are psychological harm as a result of use of the HIV self-test (for example, if participants test positive for HIV and are not prepared for the results). Although UNAIDS has recently indicated no evidence of serious adverse events related to HIV self-testing (i.e., self-harm, violence, or human rights violations)[28], occurrence of any such events are reported to all principal investigators and the Scientific Oversight Committee (SOC) within 24 hours of study staff becoming aware of them. Other adverse events include coercive testing or unintentional or unauthorized disclosure of HIV status, which are similarly reported within 24 hours.

Statistical Analysis

The primary analysis will be intention-to-treat (ITT). The primary analysis for the quantitative data will involve a mixed-effects multilevel logistic regression model to account for clustering by peer educator group and study site (participants nested in peer educators nested in study sites). These models will include an indicator term for study arm (fixed distribution, direct distribution, or control). Outcomes, including past-month

For peer review only

HIV testing and correct knowledge of status at four months, will be modeled as dichotomous variables.

Qualitative Data Analysis

Qualitative data will be analyzed using an iterative descriptive approach to characterize and describe the data at their natural level. After all interviews are transcribed and translated, a codebook will be finalized and transcripts will be coded in Dedoose, a cloud-based platform for mixed methods research.

Sample Size Considerations

The sample size calculation was based on the primary endpoint, testing for HIV in the past month assessed at the one-month visit, and the secondary endpoint, proportion of participants who correctly know their status at the four month visit. Power calculations were performed using methods for cluster randomized trials, with clustering by peer educator identity. We assume that 50% of participants will have tested in the past month in the control arm based on previous estimates of HIV testing behavior among FSW in Livingstone and Chirundu.[29,30] We estimate that in the control group, approximately 70% of participants will correctly identify their HIV status, assuming that the majority of those whose status is unknown will report that they are negative, but that approximately half of these individuals will actually be positive. Assuming 20% loss to follow-up and with 50 peer educators (clusters) per arm, a sample size of 300 participants per arm (900 total) will yield 89% power to detect a risk ratio of 1.30 for recent testing and 94% power

For peer review only

For peer review only - http://bmjopen.bmj.com/site/about/guidelines.xhtml

to detect a risk ratio of 1.20 for correct status knowledge, assuming a type I error probability of 0.05 and an intracluster correlation (ICC) of 0.03.

Confidentiality

To protect participants' confidentiality, no identifying information will be collected during any quantitative or qualitative assessment and all collected data are de-identified. A unique study identification number will be used to link data from an individual participant. Participant identity codes are kept completely separately from electronic data, in a locked cabinet in study offices. The code will be destroyed upon completion of the study.

Data Management

Quantitative data are collected directly on a study tablet via CommCare (Dimagi, Cambridge, MA), a HIPAA-compliant, cloud-based platform for electronic data collection. Qualitative data are collected via audio recorder, which are transcribed and translated verbatim. Qualitative audio recordings are destroyed upon completion of transcription and translation.

Dissemination Plan

The results of this study will be disseminated to community, policy, and scientific stakeholders. A results community meeting will be planned upon completion of the study at each study site, during which the results of the study will be announced. We will also hold a meeting with the Ministry of Health in Lusaka to disseminate our findings, and

For peer review only - http://bmjopen.bmj.com/site/about/guidelines.xhtml

submit a report detailing the findings of the study to inform policy. Finally, results will be shared with the scientific community via presentation at international conferences and journal publication.

DISCUSSION

Despite the high risk and large burden of HIV among FSW, HIV testing and HIV status knowledge in this population remain far from universal. A number of complex barriers at multiple levels can limit access to HIV testing for FSW, including fear of stigma and abuse, fear of loss of livelihood, and the financial and time costs of utilizing traditional HIV testing services. HIVST is a promising alternative HIV testing option for FSW, because it allows for a high degree of privacy, efficiency, and flexibility in HIV testing. The overarching goal of the ZEST trial is to provide robust evidence for the development of policy for implementation and scale-up of HIVST in Zambia with a specific focus on ensuring this technology is accessible to FSW. Working in concert with the Ministry of Health in Zambia and the World Health Organization, this trial was specifically designed to fill a crucial gap in the evidence by answering two questions: 1) is HIVST an acceptable alternative to standard HIV testing for FSW, and 2) will FSW access HIVST.

Question One is addressed through the direct distribution arm. In this arm, we will be able to assess whether individuals directly given a test actually use the test. By directly giving individuals the test, we are assessing best-case scenario uptake, where there are as few barriers as possible for individuals to access the test kit. The uptake results from this arm of the study will give robust evidence on whether HIVST is an

For peer review only - http://bmjopen.bmj.com/site/about/guidelines.xhtml

acceptable alternative to standard and currently-available HIV testing services. Coupled with qualitative data, we will be able to measure both the uptake of HIVST, but also participant attitudes towards HIVST. This will lead to an in-depth understanding of why participants did or did not use the test, and how participants view the role of HIVST for HIV testing in the future.

Although uptake and acceptability of HIVST is an important piece of evidence for HIVST policy, the design of this study allows for measurement of a more realistic scenario, in which participants must go to a physical distribution point to collect their kit. Question Two is thus addressed through the fixed distribution arm. This arm will measure the degree to which barriers to acquiring the HIVST kit in the store affect acquisition and use of the test. This will be tested by implementing the project within existing drug stores and health posts, without changing the hours or geographical locations of existing services. The direct and fixed distribution arms will therefore give two complementary pieces of information – first, are participants using the test kits, and second, are they able to access them independently.

A major strength of this study is that it does not rely on clinic-based sampling or recruitment. A potential benefit of HIVST over existing HIV testing services is that it may allow individuals who are not currently engaged in care or who have never sought HIV testing because of barriers to accessing care to test for HIV. HIVST may therefore be a gateway to care for individuals who have previously been difficult to engage in care. By working through peer educators, rather than in clinics, we will access a different subset of the population. Peer educator programs have been used in a variety of settings with different sex worker populations to increase HIV prevention knowledge.[31-36] In

For peer review only

473 many cases, peer educators have unique access to and rapport with FSW. This study therefore is expected to enroll a diverse population of FSW that is likely more generalizable than a clinic-based sample, while simultaneously allowing access to populations that may not currently access care.

477 A major concern with HIVST is the potential for intimate partner violence, for example if sexual partners find out about HIV status through the test. All participants will be screened for intimate partner violence at baseline, one, and four months. Intimate partner violence is very common in the FSW community, and those who have experienced intimate partner violence will be eligible to participate, although the risks of participating are explained fully. Furthermore, during follow-up peer education visits, peer educators will screen for intimate partner violence, and they will also be available between visits should any issues come up. Finally, a 24-hour telephone line will be available to all participants for any issues, including intimate partner violence, and referral will be made to counseling if desired. All reports of intimate partner violence will be reviewed to determine if they are related to HIVST or trial participation. In this study, we do not offer HIVST for FSW to test with their clients. While it is possible that this could be a benefit for some participants, it could also put some at increased risk of violence. We will collect detailed quantitative and qualitative data about with whom participants used the test, if anyone. The results of this study are expected to yield evidence of the impact of HIVST on safety endpoints, in particular intimate partner violence.

494 Another safety concern with HIVST is the potential for depression, anxiety, or suicidality as a result of learning one's HIV status. Structured interviews will contain

For peer review only

screening questions for depression, and peer educators will perform a brief screening on depression, anxiety, and self-harm at each visit. Finally, participants will be instructed to call the hotline should they need to discuss their HIV result with anyone or receive counseling.

In conclusion, the results of this study are expected to inform policy aimed at implementation and scale-up of HIVST for FSW in Zambia. This study will begin to address critical gaps in the literature describing evidence of HIV testing interventions for FSW in Southern Africa. We expect the results of this trial will have policy impact resulting in increased availability of HIVST in Zambia.

For peer review only

For peer review only - http://bmjopen.bmj.com/site/about/guidelines.xhtml

DECLARATIONS

Ethical Approval

Ethical approval was obtained for the Zambian Peer Educators for HIV Self-Testing Study from the Harvard T.H. Chan School of Public Health Institutional Review Board in Boston, Massachusetts and the ERES Converge Institutional Review Board in Lusaka, Zambia. Written informed consent is obtained from all participants by research assistants and study coordinators at each study site.

Availability of Data and Material

Upon completion of the trial, de-identified data will be available through the International Initiative for Impact Evaluation (3ie)'s public data access repository on Dataverse.

Competing Interests

None to report.

Funding

The Zambian Peer Educators for HIV Self-Testing Study is funded by the International Initiative for Impact Evaluation (3ie). CEO is supported in part by the National Institute on Drug Abuse T32-DA013911 and the National Institute of Mental Health R25-MH083620. KFO is supported in part by the National Institute of Allergy and Infectious Disease T32-AI007535. TB is supported in part by the National Institute of Child Health and Human Development R01-HD084233 and the National Institute of Allergy and Infectious Diseases R01-AI124389 and R01-AI112339. The study sponsor is not

For peer review only

involved in study design, collection, management, analysis or interpretation of the data, of writing reports or in the decision to submit the report for publication.

Authors' Contributions

Conceived and designed study: CEO, KFO, MMC, KM, WN, RS, AF, LGB, GH, TB. Created tables/figures: CEO, KFO, LGB. Wrote first draft of manuscript: CEO, KFO, MMC. Read and critically revised manuscript: CEO, KFO, MMC, KM, WN, RS, AF, LGB, GH, TB.

Acknowledgements

We gratefully acknowledge feedback on and conversations related to our study design from Anna Heard, Annette Brown, and Nancy Diaz.

For peer review only

Table 1. Study inclusion/exclusion criteria

Inclusion criteria	*Exclusion criteria*
• 18 years or older at enrollment	• Less than 18 years at enrollment
• Reports exchanging sex (vaginal, oral, and/or anal) for money or goods at least once in the past month	• Has not exchanged any form of sex in the past month
• Self-reported HIV negative and no recent HIV test (<3 months) OR HIV status unknown	• Self-reported to be living with HIV<br>• Self-reported HIV negative but tested in the within the last 3 months
• Permeant residence in the study town of enrollment (Livingstone, Chirundu, Kapiri)	• Planning to move out of the geographical area within 4 months<br>• Living in the PopART catchment area (Livingstone only)
• Willing to participate in peer education sessions and study assessments over 4-month study period	• Meets inclusion criteria but does not wish to participate
	• Concurrently participating in another HIV prevention study

For peer review only - http://bmjopen.bmj.com/site/about/guidelines.xhtml

Table 2. Outline of study visits

Study Time Point	*Visit By*	*Activities*
**Recruitment**	Peer Educator	• Peer educator discusses study with potentially eligible participant and refers them to study staff
**Enrollment**	Research Assistant	• Assessment of eligibility<br>• Informed consent (if eligible)<br>• Baseline quantitative survey<br>• Baseline qualitative survey (~5%)
**Randomization**	Research Assistant	• Randomization of peer educator and their cohort of participants once all group members are enrolled
**Intervention Visit 1 (Week 0)**	Peer Educator	• Group-based intervention<br>• HIV prevention counseling and distribution of condoms<br>• Training on HIV self-test use (in HIVST arms)<br>• Distribution of HIV self-tests or coupons (in HIVST arms)
**Intervention Visit 2 (Week 2-3)**	Peer Educator	• Screening for adverse events<br>• Discussion of any difficulty with HIV self test use (in HIVST arms)<br>• Referral to care and standard HIV testing<br>• Distribution of condoms
**One Month Visit (Week 4-5)**	Research Assistant	• One month quantitative survey<br>• One month qualitative survey (~5%)<br>• Screening for adverse events
**Intervention Visit 3 (Week 6-7)**	Peer Educator	• Screening for adverse events<br>• Discussion of any difficulty with HIV self test use (in HIVST arms)<br>• Referral to care and standard HIV testing<br>• Distribution of condoms

For peer review only - http://bmjopen.bmj.com/site/about/guidelines.xhtml

**Intervention Visit 4 (Week 10-12)**	Peer Educator	• Screening for adverse events<br>• Discussion of any difficulty with HIV self test use (in HIVST arms)<br>• Referral to care and standard HIV testing<br>• Distribution of condoms<br>• Distribution of second HIV self-test or coupon (in HIVST arms)
**Four Month Visit (Week 16-18)**	Research Assistant	• Four month quantitative survey<br>• Four month qualitative survey (~5%)<br>• Screening for adverse events<br>• HIV status assessment

For peer review only

For peer review only - http://bmjopen.bmj.com/site/about/guidelines.xhtml

Table 3. Summary of study endpoints

Endpoint	Operationalization
Primary Effectiveness Endpoint	
Tested for HIV in the past month	▪ Recent HIV testing measured by asking participants when they last tested and where (in all arms of the study)
Secondary Effectiveness Endpoints	
Use of HIV self-test	▪ Measured by buying back unused HIV self-tests at four-month visit
Awareness of HIV status	▪ Measured using a three-step approach: 1) asking participants if they know what their status is; 2) offering participants a small financial gift if they can correctly tell the interviewer what their HIV status is; 3) confirming HIV status with a rapid test.
Linkage to care and confirmatory testing	▪ Measured in two ways: 1) during follow-up visits, asking participants if they received confirmatory testing and linked to care and 2) collection of referral cards linking individual HIV self-tests to individuals via a unique identification number
Sexual behaviors	▪ Measured via computer-assisted personal interview (CAPI), including number of sexual behaviors, event-level sexual behavior data, and condom use with primary and casual partners
Safety Endpoints	
Misuse of HIV self-tests	▪ Including difficulty conducting the test (i.e., mistakes in taking the test, incorrect use of components of the test), difficulty reading the test<br>▪ Identified through interview and ongoing consultation with peer educators
Intimate partner violence	▪ Measured through surveillance and interviews by research assistants<br>▪ Any intimate partner violence (including verbal, physical, or sexual) will be documented and reported

For peer review only

For peer review only - http://bmjopen.bmj.com/site/about/guidelines.xhtml

FIGURE LEGENDS

Figure 1. Flow diagram of study enrollment, randomization, and intervention arms

Figure 2. Map of study sites in Zambia. The black star indicates Lusaka, the site of study coordination and headquarters. The circles indicate locations of study sites, including Kapiri (blue), Chirundu (green), and Livingstone (red).

Figure 3. SPIRIT flowchart of study assessments.

For peer review only

For peer review only - http://bmjopen.bmj.com/site/about/guidelines.xhtml

REFERENCES

1 Baral S, Beyrer C, Muessig K, *et al.* Burden of HIV among female sex workers in low-income and middle-income countries: a systematic review and meta-analysis. *The Lancet Infectious Diseases* 2012;**12**:538–49. doi:10.1016/S1473-3099(12)70066-X

2 Shannon K, Strathdee SA, Goldenberg SM, *et al.* Global epidemiology of HIV among female sex workers: influence of structural determinants. *The Lancet* 2015;**385**:55–71. doi:10.1016/S0140-6736(14)60931-4

3 Prüss-Ustün A, Wolf J, Driscoll T, *et al.* HIV Due to Female Sex Work: Regional and Global Estimates. *PLoS ONE* 2013;**8**:e63476–7. doi:10.1371/journal.pone.0063476

4 Baral S, Logie CH, Grosso A, *et al.* Modified social ecological model: a tool to guide the assessment of the risks and risk contexts of HIV epidemics. *BMC Public Health* 2013;**13**:482. doi:10.1186/1471-2458-13-482

5 Bekker L-G, Johnson L, Cowan F, *et al.* Combination HIV prevention for female sex workers: what is the evidence? *The Lancet* 2015;**385**:72–87. doi:10.1016/S0140-6736(14)60974-0

6 Deering KN, Lyons T, Feng CX, *et al.* Client demands for unsafe sex: The socioeconomic risk environment for HIV among street and off-street sex workers. *JAIDS Journal of Acquired Immune Deficiency Syndromes* 2013;**63**:522–31. doi:10.1097/QAI.0b013e3182968d39

7 UNAIDS. 90-90-90: An ambitious treatment target to help end the AIDS epidemic. 2014.

8 Haber N, Naidu K, Pillay D, *et al.* HIV system assessment with longitudinal treatment cascade in Kwazulu-Natal, South Africa. San Diego, CA: 2015.

9 King EJ, Maman S, Bowling JM, *et al.* The Influence of Stigma and Discrimination on Female Sex Workers' Access to HIV Services in St. Petersburg, Russia. *AIDS Behav* 2013;**17**:2597–603. doi:10.1007/s10461-013-0447-7

10 Herek GM. Confronting sexual stigma and prejudice: Theory and practice. *Journal of Social Issues* 2007;**63**:905–25.

11 Krause J, Subklew-Sehume F, Kenyon C, *et al.* Acceptability of HIV self-testing: a systematic literature review. *BMC Public Health* 2013;**13**:1–1. doi:10.1186/1471-2458-13-735

12 Pant Pai N, Sharma J, Shivkumar S, *et al.* Supervised and Unsupervised Self-Testing for HIV in High- and Low-Risk Populations: A Systematic Review. *PLoS Medicine* 2013;**10**:e1001414. doi:10.1371/journal.pmed.1001414.s005

For peer review only

13 Figueroa C, Johnson C, Verster A, *et al.* Attitudes and Acceptability on HIV Self-testing Among Key Populations: A Literature Review. *AIDS Behav* 2015;:1–17. doi:10.1007/s10461-015-1097-8

14 van Rooyen H, Tulloch O, Mukoma W, *et al.* What are the constraints and opportunities for HIVST scale-up in Africa? Evidence from Kenya, Malawi and South Africa. *J Int AIDS Soc* 2015;**18**. doi:10.7448/IAS.18.1.19445

15 Myers JE, El-Sadr WM, Zerbe A, *et al.* Rapid HIV self-testing: long in coming but opportunities beckon. *AIDS* 2013;**27**:1687–95. doi:10.1097/QAD.0b013e32835fd7a0

16 Jamil MS, Prestage G, Fairley CK, *et al.* Rationale and design of FORTH: a randomised controlled trial assessing the effectiveness of HIV self-testing in increasing HIV testing frequency among gay and bisexual men. *BMC Infectious Diseases* 2015;:1–10. doi:10.1186/s12879-015-1300-6

17 Katz D, Golden M, Hughes J, *et al.* HIV self-testing increases HIV testing frequency among high-risk men who have sex with men: a randomized controlled trial. 2015.

18 Marley G, Kang D, Wilson EC, *et al.* Introducing rapid oral–fluid HIV testing among high risk populations in Shandong, China: feasibility and challenges. *BMC Public Health* 2014;**14**:422–7. doi:10.1186/1471-2458-14-422

19 Funnell SC, Rogers PJ. *Purposeful Program Theory: Effective Use of Theories of Change and Logic Models*. San Francisco, CA: : Jossey-Bass 2011.

20 Hayes R, Ayles H, Beyers N, *et al.* HPTN 071 (PopART): Rationale and design of a cluster-randomised trial of the population impact of an HIV combination prevention intervention including universal testing and treatment – a study protocol for a cluster randomised trial. *Trials* 2014;**15**:1–17.

21 Gual A, Segura L, Contel M, *et al.* AUDIT-3 and AUDIT-4: Effectiveness of two short ofrms of the alcohol use disorders identification test. *Alcohol and Alcoholism* 2002;**37**:591–6.

22 Kroenke K, Spitzer RL, Williams JB. The PHQ-9: Validity of a brief depression severity measure. *J GEN INTERN MED* 2001;**16**:606–13.

23 Luszczynska A, Scholz U, Schwarzer R. The General Self-Efficacy Scale: Multicultural Validation Studies. *The Journal of Psychology* 2005;**139**:439–57. doi:10.3200/JRLP.139.5.439-457

24 Beattie T, Mohan HL, Bhattacharjee P, *et al.* Community Mobilization and Empowerment of Female Sex Workers in Karnataka State, South India: Associations With HIV and Sexually Transmitted Infection Risk. *American Journal of Public Health* 2014;**104**:1516–25. doi:10.2105/AJPH.2014.301911)

For peer review only - http://bmjopen.bmj.com/site/about/guidelines.xhtml

25 Stadler J, Scorgie F, van der Straten A, *et al.* Adherence and the Lie in a HIV Prevention Clinical Trial. *Medical Anthropology* 2015;:1–14. doi:10.1080/01459740.2015.1116528

26 Oldenburg C, Biello KB, Colby D, *et al.* Stigma related to sex work among men who engage in transactional sex with men in Ho Chi Minh City, Vietnam. *Int J Public Health* 2014;**59**:833–40. doi:10.1007/s00038-014-0562-x

27 Kalichman SC, Simbayi LC, Jooste S, *et al.* Development of a Brief Scale to Measure AIDS-Related Stigma in South Africa. *AIDS Behav* 2005;**9**:135–43. doi:10.1007/s10461-005-3895-x

28 A short technical update on self-testing for HIV. UNAIDS 2014.

29 Corridors of Hope Southern Africa. HIV prevention needs and opportunities in four border towns. Family Health International 2005.

30 Family Health International. Female sex workers in Border and Transportation Routes with Trend Analysis, 2000-2006. 2006;:1–79.

31 Onyango MA, Adu-Sarkodie Y, Agyarko-Poku T, *et al.* "It's All About Making a Life": Poverty, HIV, Violence, and Other Vulnerabilities Faced by Young Female Sex Workers in Kumasi, Ghana. *JAIDS Journal of Acquired Immune Deficiency Syndromes* 2016;**68**:S131–7.

32 Ford K, Wirawan DN, Suastina W, *et al.* Evaluation of a peer education programme for female sex workers in Bali, Indonesia. *Int J STD AIDS* 2008;**11**:731–3.

33 Oldenburg CE, Biello KB, Colby D, *et al.* Engagement with Peer Health Educators Is Associated with Willingness to Use Pre-Exposure Prophylaxis Among Male Sex Workers in Ho Chi Minh City, Vietnam. *AIDS Patient Care and STDs* 2014;**28**:109–12. doi:10.1089/apc.2013.0372

34 Aung T, Paw E, Aye NM, *et al.* Coverage of HIV Prevention Services for Female Sex Workers in Seven Cities of Myanmar. *AIDS Behav* 2013;**18**:37–41. doi:10.1007/s10461-013-0523-z

35 Rachakulla HK, Kodavalla V, Rajkumar H, *et al.* Condom use and prevalence of syphilis and HIV among female sex workers in Andhra Pradesh, India – following a large-scale HIV prevention intervention. 2011;:1–16. doi:10.1186/1471-2458-11-S6-S1

36 Geibel S, King'ola N, Temmerman M, *et al.* The impact of peer outreach on HIV knowledge and prevention behaviours of male sex workers in Mombasa, Kenya. *Sexually Transmitted Infections* 2012;**88**:357–62. doi:10.1136/sextrans-2011-050224

For peer review only

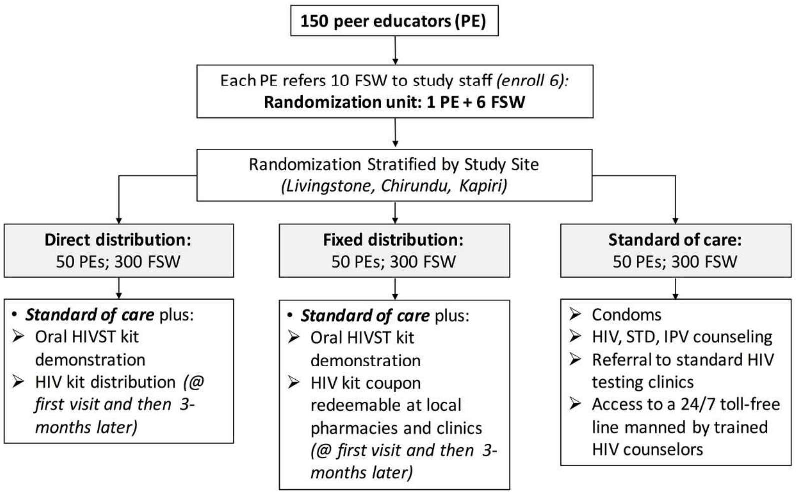

Figure 1. Flow diagram of study enrollment, randomization, and intervention arms

190x107mm (300 x 300 DPI)

For peer review only

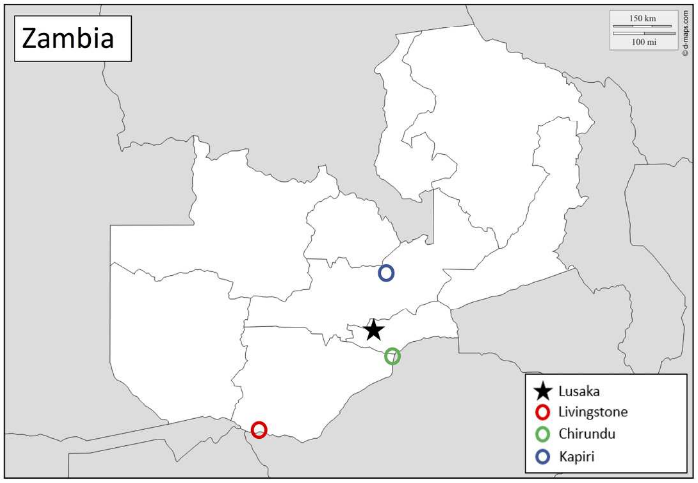

Figure 2. Map of study sites in Zambia. The black star indicates Lusaka, the site of study coordination and headquarters. The circles indicate locations of study sites, including Kapiri (blue), Chirundu (green), and Livingstone (red).

190x107mm (300 x 300 DPI)

For peer review only

Figure 3. SPIRIT flowchart of study assessments

	STUDY PERIOD						
	Enrolment	Allocation	Post-Allocation				Close-Out
**TIMEPOINT****		**0**	***2 weeks***	***1 month***	***6 weeks***	***10 weeks***	***4 months***
**ENROLMENT:**							
**Eligibility screen**	X						
**Informed consent**	X						
**Allocation**		X					
**INTERVENTIONS:**							
HIVST Kit/Coupon Distribution		X				X	
Peer Educator Visit		X	X		X	X	
**ASSESSMENTS:**							
Demographics, HIV testing history, sexual behaviors, reproductive health, HIV knowledge, empowerment	X						
HIV testing history, sexual behaviors, HIV knowledge, empowerment				X			X
HIV status knowledge							X
HIV self-test kit use							X
Adverse Events			X	X	X	X	X

Figure 3. SPIRIT flowchart of study assessments.

279x361mm (300 x 300 DPI)

SPIRIT

STANDARD PROTOCOL ITEMS: RECOMMENDATIONS FOR INTERVENTIONAL TRIALS

SPIRIT 2013 Checklist: Recommended items to address in a clinical trial protocol and related documents*

Section/item	Item No	Description	Addressed on page number
**Administrative information**			
Title	1	Descriptive title identifying the study design, population, interventions, and, if applicable, trial acronym	________1______
Trial registration	2a	Trial identifier and registry name. If not yet registered, name of intended registry	________3______
	2b	All items from the World Health Organization Trial Registration Data Set	______________
Protocol version	3	Date and version identifier	______n/a______
Funding	4	Sources and types of financial, material, and other support	________26______
Roles and responsibilities	5a	Names, affiliations, and roles of protocol contributors	________1, 27____
	5b	Name and contact information for the trial sponsor	________26______
	5c	Role of study sponsor and funders, if any, in study design; collection, management, analysis, and interpretation of data; writing of the report; and the decision to submit the report for publication, including whether they will have ultimate authority over any of these activities	_________27_____
	5d	Composition, roles, and responsibilities of the coordinating centre, steering committee, endpoint adjudication committee, data management team, and other individuals or groups overseeing the trial, if applicable (see Item 21a for data monitoring committee)	______9_______

For peer review only - http://bmjopen.bmj.com/site/about/guidelines.xhtml

**Introduction**			
Background and rationale	6a	Description of research question and justification for undertaking the trial, including summary of relevant studies (published and unpublished) examining benefits and harms for each intervention	_____5-8_______
	6b	Explanation for choice of comparators	____7_________
Objectives	7	Specific objectives or hypotheses	_____9________
Trial design	8	Description of trial design including type of trial (eg, parallel group, crossover, factorial, single group), allocation ratio, and framework (eg, superiority, equivalence, noninferiority, exploratory)	_____7________
**Methods: Participants, interventions, and outcomes**			
Study setting	9	Description of study settings (eg, community clinic, academic hospital) and list of countries where data will be collected. Reference to where list of study sites can be obtained	______10_______
Eligibility criteria	10	Inclusion and exclusion criteria for participants. If applicable, eligibility criteria for study centres and individuals who will perform the interventions (eg, surgeons, psychotherapists)	_____11________
Interventions	11a	Interventions for each group with sufficient detail to allow replication, including how and when they will be administered	_____13-14_____
	11b	Criteria for discontinuing or modifying allocated interventions for a given trial participant (eg, drug dose change in response to harms, participant request, or improving/worsening disease)	_____n/a_______
	11c	Strategies to improve adherence to intervention protocols, and any procedures for monitoring adherence (eg, drug tablet return, laboratory tests)	______n/a______
	11d	Relevant concomitant care and interventions that are permitted or prohibited during the trial	_____n/a______
Outcomes	12	Primary, secondary, and other outcomes, including the specific measurement variable (eg, systolic blood pressure), analysis metric (eg, change from baseline, final value, time to event), method of aggregation (eg, median, proportion), and time point for each outcome. Explanation of the clinical relevance of chosen efficacy and harm outcomes is strongly recommended	___16-19_______

For peer review only - http://bmjopen.bmj.com/site/about/guidelines.xhtml

Participant timeline	13	Time schedule of enrolment, interventions (including any run-ins and washouts), assessments, and visits for participants. A schematic diagram is highly recommended (see Figure)	__Table 2_______
Sample size	14	Estimated number of participants needed to achieve study objectives and how it was determined, including clinical and statistical assumptions supporting any sample size calculations	___20__________
Recruitment	15	Strategies for achieving adequate participant enrolment to reach target sample size	___20__________
**Methods: Assignment of interventions (for controlled trials)**			
Allocation:			
Sequence generation	16a	Method of generating the allocation sequence (eg, computer-generated random numbers), and list of any factors for stratification. To reduce predictability of a random sequence, details of any planned restriction (eg, blocking) should be provided in a separate document that is unavailable to those who enrol participants or assign interventions	___11__________
Allocation concealment mechanism	16b	Mechanism of implementing the allocation sequence (eg, central telephone; sequentially numbered, opaque, sealed envelopes), describing any steps to conceal the sequence until interventions are assigned	___11__________
Implementation	16c	Who will generate the allocation sequence, who will enrol participants, and who will assign participants to interventions	___11__________
Blinding (masking)	17a	Who will be blinded after assignment to interventions (eg, trial participants, care providers, outcome assessors, data analysts), and how	___n/a________
	17b	If blinded, circumstances under which unblinding is permissible, and procedure for revealing a participant's allocated intervention during the trial	__n/a_________
**Methods: Data collection, management, and analysis**			
Data collection methods	18a	Plans for assessment and collection of outcome, baseline, and other trial data, including any related processes to promote data quality (eg, duplicate measurements, training of assessors) and a description of study instruments (eg, questionnaires, laboratory tests) along with their reliability and validity, if known. Reference to where data collection forms can be found, if not in the protocol	__14-16________

	18b	Plans to promote participant retention and complete follow-up, including list of any outcome data to be collected for participants who discontinue or deviate from intervention protocols	_n/a__________
Data management	19	Plans for data entry, coding, security, and storage, including any related processes to promote data quality (eg, double data entry; range checks for data values). Reference to where details of data management procedures can be found, if not in the protocol	__14-15________
Statistical methods	20a	Statistical methods for analysing primary and secondary outcomes. Reference to where other details of the statistical analysis plan can be found, if not in the protocol	__19___________
	20b	Methods for any additional analyses (eg, subgroup and adjusted analyses)	__19 __________
	20c	Definition of analysis population relating to protocol non-adherence (eg, as randomised analysis), and any statistical methods to handle missing data (eg, multiple imputation)	__n/a__________
**Methods: Monitoring**			
Data monitoring	21a	Composition of data monitoring committee (DMC); summary of its role and reporting structure; statement of whether it is independent from the sponsor and competing interests; and reference to where further details about its charter can be found, if not in the protocol. Alternatively, an explanation of why a DMC is not needed	__9___________
	21b	Description of any interim analyses and stopping guidelines, including who will have access to these interim results and make the final decision to terminate the trial	__10___________
Harms	22	Plans for collecting, assessing, reporting, and managing solicited and spontaneously reported adverse events and other unintended effects of trial interventions or trial conduct	___19__________
Auditing	23	Frequency and procedures for auditing trial conduct, if any, and whether the process will be independent from investigators and the sponsor	__n/a_________
**Ethics and dissemination**			
Research ethics approval	24	Plans for seeking research ethics committee/institutional review board (REC/IRB) approval	___26__________

For peer review only - http://bmjopen.bmj.com/site/about/guidelines.xhtml

Protocol amendments	25	Plans for communicating important protocol modifications (eg, changes to eligibility criteria, outcomes, analyses) to relevant parties (eg, investigators, REC/IRBs, trial participants, trial registries, journals, regulators)	__n/a_________
Consent or assent	26a	Who will obtain informed consent or assent from potential trial participants or authorised surrogates, and how (see Item 32)	__26___________
	26b	Additional consent provisions for collection and use of participant data and biological specimens in ancillary studies, if applicable	__n/a__________
Confidentiality	27	How personal information about potential and enrolled participants will be collected, shared, and maintained in order to protect confidentiality before, during, and after the trial	___21_________
Declaration of interests	28	Financial and other competing interests for principal investigators for the overall trial and each study site	___26__________
Access to data	29	Statement of who will have access to the final trial dataset, and disclosure of contractual agreements that limit such access for investigators	__26_________
Ancillary and post-trial care	30	Provisions, if any, for ancillary and post-trial care, and for compensation to those who suffer harm from trial participation	___n/a________
Dissemination policy	31a	Plans for investigators and sponsor to communicate trial results to participants, healthcare professionals, the public, and other relevant groups (eg, via publication, reporting in results databases, or other data sharing arrangements), including any publication restrictions	__21_________
	31b	Authorship eligibility guidelines and any intended use of professional writers	___n/a________
	31c	Plans, if any, for granting public access to the full protocol, participant-level dataset, and statistical code	___26__________
**Appendices**			
Informed consent materials	32	Model consent form and other related documentation given to participants and authorised surrogates	__n/a_________
Biological specimens	33	Plans for collection, laboratory evaluation, and storage of biological specimens for genetic or molecular analysis in the current trial and for future use in ancillary studies, if applicable	__n/a_________

For peer review only - http://bmjopen.bmj.com/site/about/guidelines.xhtml

*It is strongly recommended that this checklist be read in conjunction with the SPIRIT 2013 Explanation & Elaboration for important clarification on the items. Amendments to the protocol should be tracked and dated. The SPIRIT checklist is copyrighted by the SPIRIT Group under the Creative Commons "Attribution-NonCommercial-NoDerivs 3.0 Unported" license.

For peer review only

For peer review only - http://bmjopen.bmj.com/site/about/guidelines.xhtml
